# The efficacy and safety of combined traditional Chinese and Western medicine treatment for PCOS-related infertility: an umbrella meta-analysis

**DOI:** 10.3389/fmed.2026.1877844

**Published:** 2026-08-06

**Authors:** Hongwen Li, Chao Sun, Qingchen Zhou, Guangzhong Du

**Affiliations:** 1 College of Acupuncture and Tuina, Shandong University of Traditional Chinese Medicine, Jinan, Shandong, China; 2 Department of Acupuncture and Tuina, Qilu Hospital of Shandong University, Jinan, Shandong, China

**Keywords:** acupuncture, Chinese herbal medicine, infertility, integrated traditional Chinese and western medicine, polycystic ovary syndrome, umbrella meta-analysis

## Abstract

**Background:**

Polycystic ovary syndrome (PCOS) commonly causes infertility in women of reproductive age. Integrated Chinese–Western medicine is used to improve reproductive outcomes and reduce adverse effects, but its reliability remains uncertain.

**Methods:**

Efficacy and safety meta-analyses of integrated traditional Chinese medicine (TCM) and Western medicine for PCOS-related infertility were synthesized. PubMed, Embase, Web of Science, the Cochrane Library, the China National Knowledge Infrastructure (CNKI), Wanfang, and the Chinese Science and Technology Periodical Database (VIP) were searched from inception to November 2025. A Measurement Tool to Assess Systematic Reviews (AMSTAR) and Grading of Recommendations Assessment, Development and Evaluation (GRADE) assessed quality and certainty. Outcomes were classified by significance and evidence strength.

**Results:**

Twenty meta-analyses included 39 intervention types and 153 outcomes, 135 outcomes (36 interventions) favored integrated treatment, and 18 (13 interventions) were non-significant. Heterogeneity and publication bias affected 33 and 48.4%, respectively. AMSTAR scores were 4–9. Four outcomes from two interventions met Class I-III criteria. Chinese medicine plus Western medicine using the TCM Cycle Method showed Class I evidence for pregnancy rate (OR: 2.76, 95% CI: 2.43, 3.13) and Class II evidence for total clinical efficiency (OR: 3.78, 95% CI: 3.29, 4.34) and ovulation rate (OR: 2.90, 95% CI: 2.39, 3.50). TCM cycle therapy plus ovulation induction showed Class III evidence for ovulation rate (RR: 1.10, 95% CI: 1.06, 1.14). GRADE ratings were high for 45, moderate for 92, and low for 16 outcomes.

**Conclusion:**

Integrated therapy showed promising associations, but robust evidence remains limited; only four of 153 outcomes met Class I-III criteria. Rigorous randomized trials are required.

**Systematic review registration:**

https://www.crd.york.ac.uk/PROSPERO/view/CRD420251275827, Identifier: CRD420251275827.

## Introduction

Polycystic ovary syndrome (PCOS), the most common endocrine disease in women of reproductive age, is a cause of anovulatory infertility (1, 2). According to epidemiological evidence, the range of prevalence is approximately 5–20%, and this range depends on the diagnostic criteria and the nature of the population (3). PCOS is clinically manifested by hyperandrogenism, ovulatory abnormality, and polycystic ovaries. Insulin resistance and obesity are also very often associated with it (4, 5). In addition to reproductive impairment, PCOS is linked with a higher risk of adverse pregnancy outcomes, such as miscarriage, gestational diabetes, and high blood pressure disorders, and this gives PCOS a significant global health and socioeconomic burden (6, 7). Nevertheless, even with the international diagnostic frameworks, heterogeneity in clinical presentation and underlying pathophysiology exists and makes it a challenge to provide individualized infertility management (2, 8).

The most common method of managing infertility in women with PCOS is Western medical therapy, in which ovulation-inducing drugs, mainly letrozole and clomiphene citrate, are widely applied in clinical practice (9, 10). Even though letrozole is currently suggested as a first-line therapy, there is still a significant percentage of patients who respond poorly or become resistant to the drug. Adverse effects associated with treatment, including ovarian hyperstimulation syndrome and multiple pregnancy, have been reported (9, 11). It is due to these restrictions that considerable interest has arisen in such complementary and integrative approaches to therapy. Traditional Chinese medicine (TCM), including herbal preparations, acupuncture, and cycle-oriented treatment plans, has long been considered able to modify menstrual activity and increase fertility rates (12, 13). The experimental and clinical evidence indicates that the action of TCM-based interventions might have a multi-target effect via regulation of endocrine activity, improvement in insulin sensitivity, reduction of hyperandrogenism, and increase in endometrial receptivity (14, 15).

Over the past few years, more and more systematic reviews and meta-analyses have been carried out to determine the effectiveness and safety of integrated traditional Chinese and Western medicine in the treatment of infertility secondary to PCOS. Numerous reports have reported improvements in ovulation rates, pregnancy results, hormonal levels, and treatment safety. Nevertheless, the general conclusions are not very uniform and rather conflicting occasionally (15, 16). Such inconsistencies can be connected with methodological constraints, limited sample sizes, and variation in intervention procedures and measures of outcomes. Moreover, the evidence for various outcomes in terms of credibility and certainty has not been formally evaluated. An umbrella meta-analysis is used to combine the results of various meta-analyses in a synthesis and can enable the systematic assessment of the strength of evidence, which is an appropriate methodological choice to handle these problems (17). This design thus supports the use of an umbrella meta-analysis to determine the efficacy and safety of integrated Chinese and Western medicine for PCOS-related infertility, classify the strength of evidence by outcome, and support further clinical practice and research.

## Methods and analysis

### Design and registration

This was an umbrella meta-analysis that adhered to the Preferred Reporting Items for Overviews of Reviews (PRIOR) (18) and applicable Preferred Reporting Items for Systematic Reviews and Meta-Analyses (PRISMA) 2020 (19) items to find, screen, extract, and synthesize data using published meta-analyses of the effectiveness and safety of the combination of traditional Chinese and Western medicine for infertility due to PCOS. This was an umbrella review done in accordance with the Joanna Briggs Institute (JBI) Manual of Evidence Synthesis (20) and the Cochrane Handbook for Systematic Reviews (21). It is registered with PROSPERO (CRD420251275827). No separate peer-reviewed or publicly archived protocol was published beyond the PROSPERO registration; therefore, the PROSPERO record served as the *a priori* protocol for this umbrella review.

### Eligibility criteria

This is an umbrella meta-analysis comprising systematic reviews and meta-analyses that had quantitative syntheses limited to randomized controlled trials (RCTs). Eligible reviews were used to evaluate the efficacy and safety of integrated traditional Chinese and Western medicine in treating infertility caused by PCOS. Integrated interventions were described as therapeutic interventions involving the use of traditional Chinese medicine modalities including herbal prescriptions, acupuncture, moxibustion, or cycle-based medical methods in conjunction with Western medical treatments, which could include ovulation induction agents, assisted reproductive technologies (ART), or hormonal treatments. Comparators included Western medicine, placebo, or conventional standard care. Reviews needed to report at least one outcome related to reproductive efficacy, endocrine or metabolic parameters, endometrial characteristics, or safety of treatment. Excluded publications included narrative or scoping reviews that lacked a quantitative synthesis of their results, original research papers, study protocols, conference abstracts, animal or *in vitro* studies, or reviews assessing traditional Chinese medicine or Western medicine as an intervention per se. Reviews that did not include enough quantitative data in order to be able to determine the effect estimates, the heterogeneity, and the credibility of evidence as a whole were excluded (17).

### Population

The study population included women of reproductive age who were not pregnant and whose infertility was caused by PCOS. In the reviewed articles, PCOS was defined based on internationally agreed criteria, such as the Rotterdam criteria, the National Institutes of Health (NIH) criteria, or the Androgen Excess Society (AES) criteria (2, 7). Infertility was defined as failure to attain a clinical pregnancy following at least 12 months of regular, unprotected intercourse in line with the international standards of reproductive health (6). Women who tried spontaneous conception and those undergoing assisted reproductive methods, including ovulation induction or *in vitro* fertilization, were eligible to be included, provided that PCOS was their major cause of infertility. Articles were excluded when the primary etiology of infertility was not associated with PCOS, including isolated male-factor infertility, tubal disease, abnormal developmental anomalies of the uterus, premature ovarian insufficiency, or other endocrine disorders (e.g., thyroid dysfunction, hyperprolactinemia, or congenital adrenal hyperplasia). This definition of the population was offered with the purpose of providing clinical specificity and the ability to interpret reproductive, endocrine, endometrial, and safety outcomes associated with PCOS-related infertility.

### Exposure

This was a meta-analysis performed using an umbrella approach to evaluate the effectiveness of integrative therapeutic interventions using traditional Chinese medicine (TCM) with Western medicine (WM) in managing infertility among women with PCOS. The TCM approaches encompassed acupuncture, herbal medicines, moxibustion, and cycle-based treatment modalities, applied in combination with Western medical treatments, including ovulation induction agents, assisted reproductive technologies (ART), such as *in vitro* fertilization-embryo transfer (IVF-ET), and hormonal therapy. The outcomes that were analyzed included reproductive outcomes, which primarily included the ovulation rate and pregnancy outcomes. Endometrial functionality, insulin sensitivity, and metabolic parameters were also evaluated. The duration of exposure and treatment regimens were different across studies, which shows what is done in practice. The incorporated meta-analyses involved the comparison of the intervention of integrated TCM and WM with Western medicine, placebo, or standard conventional care alone (22–25).

### Study designs

Only systematic reviews and meta-analyses were included. These reviews tested the efficacy and safety of integrated therapeutic strategies and had to utilize standardized methodological approaches, such as comprehensive literature searches, accurate inclusion and exclusion criteria, quality assessment, and synthesis of quantitative information. Only meta-analyses that had evidence based on randomized controlled trials were included. Observational study designs were excluded from the reviews.

### Information sources

Within the scope of the present study, PubMed, Embase, Web of Science, the Cochrane Library, the Wanfang Data Knowledge Service Platform, the China National Knowledge Infrastructure (CNKI), and the Chinese Science and Technology Periodical Database (VIP) were searched to identify systematic reviews and meta-analyses on the topic of integrated traditional Chinese and Western medicine for treating infertility in patients with PCOS from inception to November 2025. To identify other potentially relevant articles that met eligibility criteria, reference lists of eligible studies were manually screened for additional records.

### Search strategy

In accordance with Scottish Intercollegiate Guidelines Network (SIGN) guidance, the databases were searched using Medical Subject Headings (MeSH) that were based on polycystic ovary syndrome and infertility. The search strategy was as follows: Polycystic Ovary Syndrome [MeSH Term] or Polycystic Ovary Syndrome [Title/Abstract], and Infertility [MeSH Term] or infertility [Title/Abstract]. Filters were used to include systematic reviews and meta-analyses as results by entering terms in the Title/Abstract field, including meta-analysis or systematic review. The search was also narrowed down to terms related to Western medicine and traditional Chinese medicine, including acupuncture, herbal medicine, ovulation induction, and assisted reproductive technologies. The description of the search strategy can be found in Supplementary Table S1.

### Study selection

**Figure 1:**
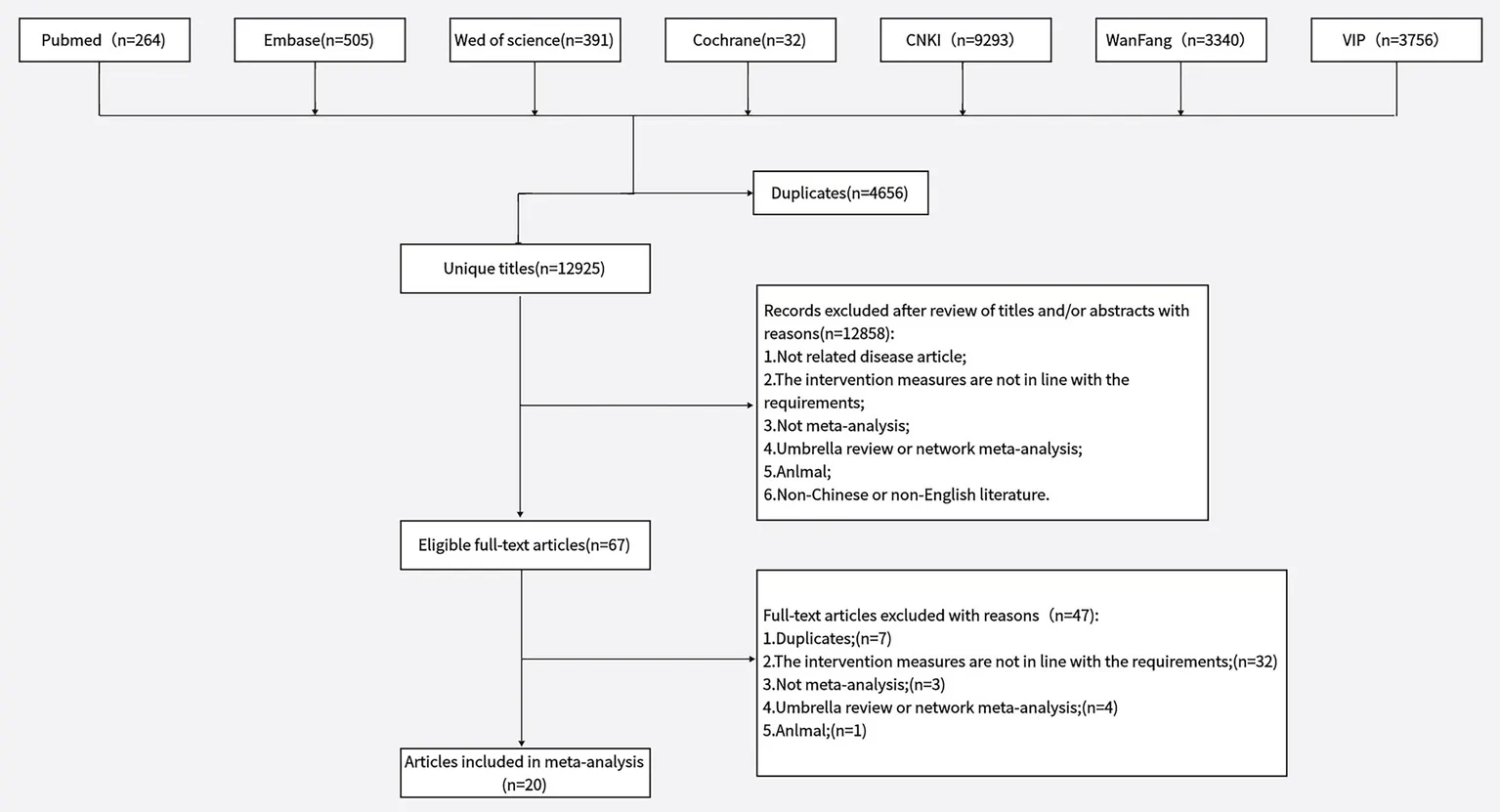
Flowchart of literature search and screening.

EndNote 21 was used to manage the literature, which was then screened after duplicate records were removed. Two authors went through titles and abstracts to assess the eligibility of meta-analyses. A third author resolved the disagreements. The reference lists of relevant studies were manually reviewed to locate other potentially eligible meta-analyses (Figure 1).

### Measurement of methodological quality

Two authors assessed each meta-analysis independently using A Measurement Tool to Assess Systematic Reviews (AMSTAR) (26, 27). The Grading of Recommendations Assessment, Development and Evaluation (GRADE) framework was used to rate the certainty of the outcomes as high, moderate, or low, based on eight domains: risk of bias, inconsistency, indirectness, imprecision, publication bias, plausible confounding, magnitude of effect, and dose–response gradient (28). These outcomes were categorized into five evidence classes, namely, Class I (convincing), Class II (highly suggestive), Class III (suggestive), Class IV (weak), and non-significant (NS) evidence (26–28) (Supplementary Table S2).

Evidence classes were assigned using prespecified criteria that considered sample size, level of statistical significance, between-study heterogeneity, and potential publication bias. The sample-size criterion was met when an outcome included more than 1,000 cases, or more than 20,000 participants for continuous variables. For statistical significance, Class I and Class II evidence required *p* < 10^−6^, whereas Class III evidence required *p* < 0.001. Class IV evidence referred to other statistically significant associations with *p* < 0.05. Outcomes with *p* > 0.05 were regarded as non-significant. Heterogeneity was evaluated using the *I*^2^ statistic, and *I*^2^ values below 50% were considered to indicate the absence of substantial heterogeneity. Publication bias was examined using Egger’s test, with *p* < 0.05 suggesting possible publication bias.

### Data extraction

Data were extracted by two scientists working independently from eligible studies, including author details, publication date, outcomes reported, the size of the study, case number, total sample size, study design, and effect estimate with their corresponding 95% confidence intervals. Meta-analytic statistics, measures of heterogeneity (*I*^2^and Cochran’s *Q*), and tests of publication bias (Egger’s test, Begg’s test, and funnel plots) were recorded. Where dose–response analysis or subgroup analysis had been presented, the subgroup estimates and subgroup non-linearity *p*-values were extracted. A third investigator resolved the disagreements.

To assess potential overlap among the included meta-analyses, we constructed a citation matrix to present whether the same primary studies were included in more than one meta-analysis. This matrix was used to improve the transparency of overlapping evidence across the included reviews (Supplementary Table S3).

### Data summary

Effect estimates, including risk ratios (RRs), odds ratios (ORs), pooled odds ratios (PORs), mean differences (MDs), and standardized mean differences (SMDs), with corresponding 95% confidence intervals (CIs), were recalculated with fixed-effects or random-effects models. Heterogeneity was assessed using *I*^2^ and Cochran’s *Q* tests. Egger’s or Begg’s tests were applied to meta-analyses containing over 10 studies to assess small-study effects when the data were available. In the case of Class I and Class II evidence, sensitivity analysis was performed to estimate the effect of individual studies on statistical significance. The included meta-analyses were used to extract dose–response analyses on pregnancy rate, ovulation rate, menstrual improvement, and endometrial thickness. In instances where the latest meta-analysis literature did not encompass clinical studies identified in previous reviews, such studies were re-extracted and recalculated. A *p*-value of < 0.10 was considered statistically significant heterogeneity. For other statistical tests, the threshold was *p* < 0.05. To enhance interpretability of forest plots and minimize distortion generated by large data dispersion and varying and inconsistent measurement units, MD (rather than SMD) as originally reported in the study was used for plotting.

## Results

### Characteristics of meta-analyses

**Figure 2:**
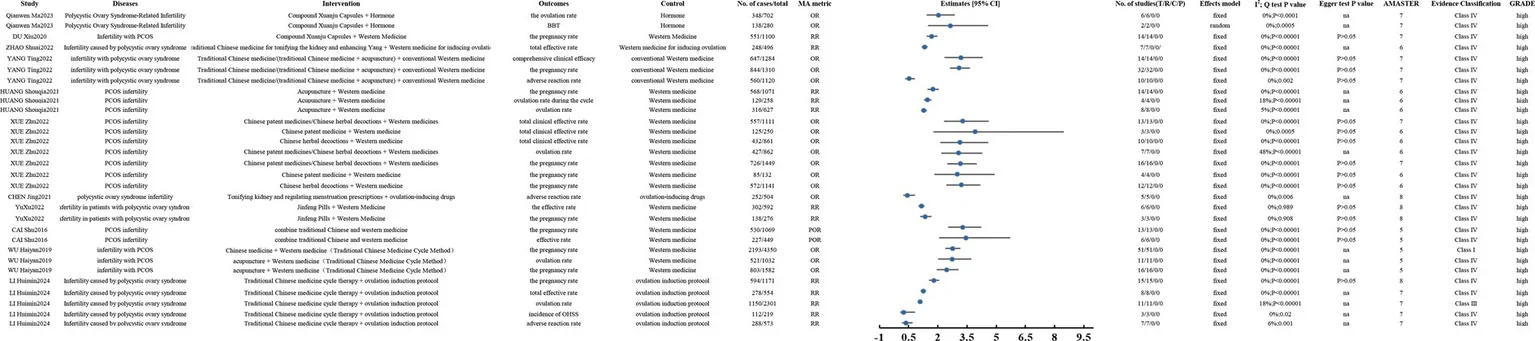
Forest plot of binary categorical variables.

**Figure 3:**
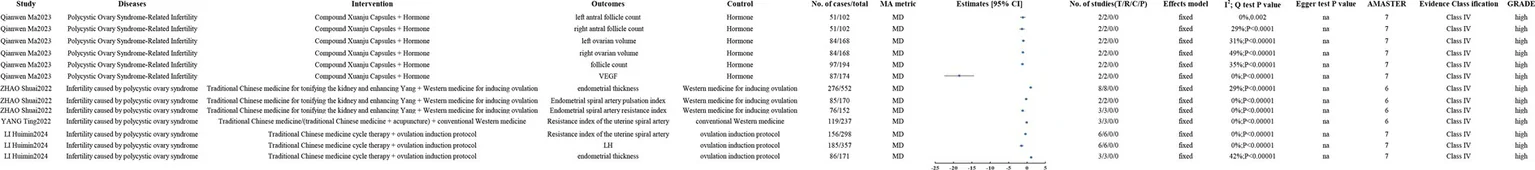
Forest plot of continuous variable.

Figure 1 shows the process of literature search and selection of the studies. A total of 17,581 unique records were identified. Among them, 20 meta-analyses (29–48) were included based on the predefined inclusion criteria. All the included meta-analyses were based on RCTs, and no meta-analysis of observational studies was found. Overall, 153 outcomes concerning PCOS-related infertility were identified, of which 135 were statistically significant and 18 were non-significant (Table 1 and Figures 2, 3). A citation matrix was added to present the overlap of primary studies across the included meta-analyses and to help readers identify potential duplicate evidence (Supplementary Table S3). Following the assessment of quality, 149 outcomes were defined as Class IV or NS. The remaining four outcomes reached Class I, II, or III evidence. The vast majority of findings were categorized as Class IV or NS, mainly because of small sample sizes (Supplementary Table S2).

**Table 1:** Study characteristic.

Disease	Intervention	Control	Total eligible MA	Outcome	Included MA	No. of cases/total	MA metric	Estimates [95% CI]	No. of studies (T/R)	Effects model	I2; Q test *P*-value	Egge*r*’s test *P*-value	AMASTER score	Evidence classification	GRADE
Significant															
Infertility with polycystic ovary syndrome	Compound Xuanju capsules +CC	CC	1	Menstrual improvement rate	Zhou Kunyan, 2014 (48)	95/190	RR	1.67 [1.30.2.14]	3/3	Fixed	0%; *p* < 0.0001	*P* < 0.05	5	Class IV	Moderate
Infertility with polycystic ovary syndrome	Compound Xuanju capsules +CC	CC	1	T	Zhou Kunyan, 2014 (48)	30/60	MD	-0.34 [−0.40,-0.28]	1/1	Fixed	na; *P* < 0.0001	*P* < 0.05	5	Class IV	Moderate
Infertility with polycystic ovary syndrome	Compound Xuanju capsules +CC	CC	1	Ovulation rate during the cycle	Zhou Kunyan, 2014 (48)	122/249	RR	1.23 [1.04,1.45]	1/1	Fixed	na; 0.02	*P* < 0.05	5	Class IV	Moderate
Infertility with polycystic ovary syndrome	Compound Xuanju capsules +CC	CC	1	Menstrual improvement rate	Zhou Kunyan, 2014 (48)	36/54	RR	2.00 [1.36,2.94]	1/1	Fixed	na; 0.0004	*P* < 0.05	5	Class IV	Moderate
Infertility with polycystic ovary syndrome	Compound Xuanju capsules +LE	LE	1	BMI	Zhou Kunyan, 2014 (48)	45/90	MD	–3.12 [−3.55,-2.69]	1/1	Fixed	na; *P* < 0.0001	*P* < 0.05	5	Class IV	Moderate
Polycystic ovary syndrome-related infertility	Compound Xuanju capsules + hormone	Hormone	1	The ovulation rate	Qianwen Ma, 2023 (31)	348/702	OR	2.04 [1.46,2.84]	6/6	Fixed	0%; *P* < 0.0001	na	7	Class IV	High
Polycystic ovary syndrome-related infertility	Compound Xuanju capsules + hormone	Hormone	1	The pregnancy rate	Qianwen Ma, 2023 (31)	457/915	OR	2.43 [1.84,3.21]	11/11	Fixed	0%; *p* < 0.00001	P < 0.05	8	Class IV	Moderate
Polycystic ovary syndrome-related infertility	Compound Xuanju capsules + hormone	Hormone	1	LH level	Qianwen Ma, 2023 (31)	511/1022	MD	−2.47 [−3.07,–1.86]	12/12	Random	91%; *P* < 0.00001	*P* < 0.05	8	Class IV	Low
Polycystic ovary syndrome-related infertility	Compound Xuanju capsules + hormone	Hormone	1	FSH level	Qianwen Ma, 2023 (31)	511/1022	MD	0.91 [0.32,1.49]	12/12	Random	97%;0.002	*P* < 0.05	8	Class IV	Low
Polycystic ovary syndrome-related infertility	Compound Xuanju capsules + hormone	Hormone	1	E2 level	Qianwen Ma, 2023 (31)	288/576	MD	15.78 [7.96,23.60]	7/7	Random	94%; *P* < 0.0001	*P* < 0.05	8	Class IV	Low
Polycystic ovary syndrome-related infertility	Compound Xuanju capsules + hormone	Hormone	1	LH/FSH ratio	Qianwen Ma, 2023 (31)	69/138	MD	−0.45 [−0.67,-0.22]	2/2	Random	86%; *P* < 0.0001	*P* < 0.05	8	Class IV	Low
Polycystic ovary syndrome-related infertility	Compound Xuanju capsules + hormone	Hormone	1	T level	Qianwen Ma, 2023 (31)	393/786	MD	−0.36 [−0.52,–0.20]	9/9	Random	99%; *P* < 0.0001	na	7	Class IV	Moderate
Polycystic ovary syndrome-related infertility	Compound Xuanju capsules + hormone	Hormone	1	Endometrial thickness	Qianwen Ma, 2023 (31)	233/467	MD	1.60 [0.69,2.51]	5/5	Random	97%; 0.0005	*P* < 0.05	8	Class IV	Low
Polycystic ovary syndrome-related infertility	Compound Xuanju capsules + hormone	Hormone	1	Overall rate of effective treatment	Qianwen Ma, 2023 (31)	313/626	OR	5.35 [3.22,8.89]	6/6	Fixed	0%; *P* < 0.00001	*P* < 0.05	8	Class IV	Moderate
Polycystic ovary syndrome-related infertility	Compound Xuanju capsules + hormone	Hormone	1	Overall rate of effective Chinese medicine-based treatment	Qianwen Ma, 2023 (31)	57/95	OR	4.73 [2.00,11.19]	2/2	Fixed	0%; 0.0004	*P* < 0.05	8	Class IV	Moderate
Polycystic ovary syndrome-related infertility	Compound Xuanju capsules + hormone	Hormone	1	BBT	Qianwen Ma, 2023 (31)	138/280	OR	2.65 [1.53,4.57]	2/2	Random	0%; 0.0005	na	7	Class IV	High
Polycystic ovary syndrome-related infertility	Compound Xuanju capsules + hormone	Hormone	1	Left antral follicle count	Qianwen Ma, 2023 (31)	51/102	MD	−1.13 [−1.85,-0.42]	2/2	Fixed	0%, 0.002	na	7	Class IV	High
Polycystic ovary syndrome-related infertility	Compound Xuanju capsules + hormone	Hormone	1	Right antral follicle count	Qianwen Ma, 2023 (31)	51/102	MD	−1.36 [−2.00,–0.72]	2/2	Fixed	29%; *P* < 0.0001	na	7	Class IV	High
Polycystic ovary syndrome-related infertility	Compound Xuanju capsules + hormone	Hormone	1	Left ovarian volume	Qianwen Ma, 2023 (31)	84/168	MD	−0.96 [−1.20,-0.72]	2/2	Fixed	31%; *P* < 0.00001	na	7	Class IV	High
Polycystic ovary syndrome-related infertility	Compound Xuanju capsules + hormone	Hormone	1	Right ovarian volume	Qianwen Ma, 2023 (31)	84/168	MD	−1.09 [−1.34,-0.85]	2/2	Fixed	49%; *P* < 0.00001	na	7	Class IV	High
Polycystic ovary syndrome-related infertility	Compound Xuanju capsules + hormone	Hormone	1	Follicle count	Qianwen Ma, 2023 (31)	97/194	MD	−1.23 [−1.56,-0.89]	2/2	Fixed	35%; *P* < 0.00001	na	7	Class IV	High
Polycystic ovary syndrome-related infertility	Compound Xuanju capsules + hormone	Hormone	1	Maximum follicle diameter	Qianwen Ma, 2023 (31)	86/172	MD	2.52 [0.68,4.37]	2/2	Random	79%; 0.007	na	7	Class IV	Moderate
Polycystic ovary syndrome-related infertility	Compound Xuanju capsules + hormone	Hormone	1	HGF	Qianwen Ma, 2023 (31)	87/174	MD	−85.40 [−104.97,-65.82]	2/2	Random	84%; *P* < 0.00001	na	7	Class IV	Moderate
Polycystic ovary syndrome-related infertility	Compound Xuanju capsules + hormone	Hormone	1	VEGF	Qianwen Ma, 2023 (31)	87/174	MD	−18.46 [−22.43,–14.49]	2/2	Fixed	0%; *P* < 0.00001	na	7	Class IV	High
Infertility with PCOS	Compound Xuanju capsules + Western medicine	Western Medicine	1	The pregnancy rate	Du Xiu, 2020 (36)	551/1100	RR	1.68 [1.45,1.94]	14/14	Fixed	0%; *P* < 0.00001	*P* > 0.05	7	Class IV	High
Infertility with PCOS	Compound Xuanju capsules + Western medicine	Western Medicine	1	Ovulation rate during the cycle	Du Xiu, 2020 (36)	477/965	RR	1.17 [1.03,1.34]	6/6	Random	51%; 0.02	*P* < 0.05	6	Class IV	Low
Infertility with PCOS	Compound Xuanju capsules + Western medicine	Western Medicine	1	Comparison of the improvement of TCM symptoms	Du Xiu, 2020 (36)	224/447	RR	1.60 [1.28,2.00]	6/6	Random	62%; *P* < 0.0001	*P* < 0.05	6	Class IV	Low
Infertility with PCOS	Compound Xuanju capsules + Western medicine	Western Medicine	1	T	Du Xiu, 2020 (36)	223/446	MD	−0.53 [−0.90,-0.16]	5/5	Random	99%; 0.005	*P* < 0.05	6	Class IV	Low
Infertility with PCOS	Compound Xuanju capsules + Western medicine	Western Medicine	1	Endometrial thickness	Du Xiu, 2020 (36)	245/489	MD	1.56 [1.26,1.86]	6/6	Random	70%; *P* < 0.00001	*P* < 0.05	6	Class IV	Low
Infertility caused by polycystic ovary syndrome	Traditional Chinese medicine for tonifying the kidney and enhancing Yang + Western medicine for inducing ovulation	Western medicine for inducing ovulation	1	Total effective rate	Zhao Shuai, 2022 (47)	248/496	RR	1.32 [1.19,1.46]	7/7/	Fixed	0%; *P* < 0.00001	na	6	Class IV	High
Infertility caused by polycystic ovary syndrome	Traditional Chinese medicine for tonifying the kidney and enhancing Yang + Western medicine for inducing ovulation	Western medicine for inducing ovulation	1	The pregnancy rate	Zhao Shuai, 2022 (47)	509/1001	RR	1.95 [1.66,2.28]	14/14	Fixed	0%; *P* < 0.00001	*P* < 0.05	7	Class IV	Moderate
Infertility caused by polycystic ovary syndrome	Traditional Chinese medicine for tonifying the kidney and enhancing Yang + Western medicine for inducing ovulation	Western medicine for inducing ovulation	1	Luteinizing hormone	Zhao Shuai, 2022 (47)	394/786	SMD	−1.39 [−1.78,-0.99]	11/11	Random	83%; *P* < 0.00001	na	6	Class IV	Moderate
Infertility caused by polycystic ovary syndrome	Traditional Chinese medicine for tonifying the kidney and enhancing Yang + Western medicine for inducing ovulation	Western medicine for inducing ovulation	1	Luteinizing hormone/follicle-stimulating hormone	Zhao Shuai, 2022 (47)	144/288	SMD	−1.73 [−2.18,-1.29]	5/5	Random	61%; *P* < 0.00001	na	6	Class IV	Moderate
Infertility caused by polycystic ovary syndrome	Traditional Chinese medicine for tonifying the kidney and enhancing Yang + Western medicine for inducing ovulation	Western medicine for inducing ovulation	1	T	Zhao Shuai, 2022 (47)	304/606	SMD	−1.34 [−1.93,-0.75]	8/8	Random	90%; *P* < 0.00001	na	6	Class IV	Moderate
Infertility caused by polycystic ovary syndrome	Traditional Chinese medicine for tonifying the kidney and enhancing Yang + Western medicine for inducing ovulation	Western medicine for inducing ovulation	1	Endometrial thickness	Zhao Shuai, 2022 (47)	276/552	SMD	0.97 [0.80,1.15]	8/8	Fixed	29%; *P* < 0.00001	na	6	Class IV	High
Infertility caused by polycystic ovary syndrome	Traditional Chinese medicine for tonifying the kidney and enhancing Yang + Western medicine for inducing ovulation	Western medicine for inducing ovulation	1	Endometrial spiral artery pulsatility index	Zhao Shuai, 2022 (47)	85/170	MD	−0.23 [−0.28,-0.19]	2/2	Fixed	0%; *P* < 0.00001	na	6	Class IV	High
Infertility caused by polycystic ovary syndrome	Traditional Chinese medicine for tonifying the kidney and enhancing Yang + Western medicine for inducing ovulation	Western medicine for inducing ovulation	1	Endometrial spiral artery resistance index	Zhao Shuai, 2022 (47)	76/152	SMD	−1.16 [−1.51,-0.82]	3/3	Fixed	0%; *P* < 0.00001	na	6	Class IV	High
Infertility with polycystic ovary syndrome	Traditional Chinese medicine/(traditional Chinese medicine + acupuncture) + conventional Western medicine	Conventional Western medicine	1	Comprehensive clinical efficacy	Yang Ting, 2022 (46)	647/1284	OR	3.18 [2.37,4.27]	14/14	Fixed	0%; *P* < 0.00001	*P* > 0.05	7	Class IV	High
Infertility with polycystic ovary syndrome	Traditional Chinese medicine/(traditional Chinese medicine + acupuncture) + conventional Western medicine	Conventional Western medicine	1	The pregnancy rate	Yang Ting, 2022 (46)	844/1310	OR	3.10 [2.65,3.63]	32/32	Fixed	0%; *P* < 0.00001	*P* > 0.05	7	Class IV	High
Infertility with polycystic ovary syndrome	Traditional Chinese medicine/(traditional Chinese medicine + acupuncture) + conventional Western medicine	Conventional Western medicine	1	Bad rectification rate	Yang Ting, 2022 (46)	560/1120	OR	0.53 [0.36,0.80]	10/10	Fixed	0%; 0.002	*P* > 0.05	7	Class IV	High
Infertility with polycystic ovary syndrome	Traditional Chinese medicine/(traditional Chinese medicine + acupuncture) + conventional Western medicine	Conventional Western medicine	1	The abortion rate	Yang Ting, 2022 (46)	516/1032	OR	0.33 [0.21,0.53]	7/7	Fixed	20%; *P* < 0.00001	*P* < 0.05	7	Class IV	Moderate
Infertility with polycystic ovary syndrome	Traditional Chinese medicine/(traditional Chinese medicine + acupuncture) + conventional Western medicine	Conventional Western medicine	1	The ovulation rate	Yang Ting, 2022 (46)	642/1282	OR	2.42 [1.89,3.11]	11/11	Fixed	0%; *P* < 0.00001	*P* < 0.05	7	Class IV	Moderate
Infertility with polycystic ovary syndrome	Traditional Chinese medicine/(traditional Chinese medicine + acupuncture) + conventional Western medicine	Conventional Western medicine	1	LH level	Yang Ting, 2022 (46)	492/998	MD	−2.10 [−2.32,–1.88]	13/13	Fixed	26%; *P* < 0.00001	*P* < 0.05	6	Class IV	Moderate
Infertility with polycystic ovary syndrome	Traditional Chinese medicine/(traditional Chinese medicine + acupuncture) + conventional Western medicine	Conventional Western medicine	1	LH/FSH	Yang Ting, 2022 (46)	132/265	MD	−0.70 [−0.77,–0.62]	4/4	Fixed	44%; *P* < 0.00001	*P* < 0.05	6	Class IV	Moderate
Infertility with polycystic ovary syndrome	Traditional Chinese medicine/(traditional Chinese medicine + acupuncture) + conventional Western medicine	Conventional Western medicine	1	T level	Yang Ting, 2022 (46)	450/891	MD	−0.45 [−0.53,–0.38]	9/9	Fixed	24%; *P* < 0.00001	*P* < 0.05	6	Class IV	Moderate
Infertility with polycystic ovary syndrome	Traditional Chinese medicine/(traditional Chinese medicine + acupuncture) + conventional Western medicine	Conventional Western medicine	1	Endometrial thickness	Yang Ting, 2022 (46)	426/836	MD	1.81 [1.52,2.10]	10/10	Random	82%; *P* < 0.00001	*P* > 0.05	7	Class IV	Moderate
Infertility with polycystic ovary syndrome	Traditional Chinese medicine/(traditional Chinese medicine + acupuncture) + conventional Western medicine	Conventional Western medicine	1	Endometrial spiral artery pulsatility index	Yang Ting, 2022 (46)	119/237	MD	−0.23 [−0.29,-0.16]	3/3	Fixed	33%; *P* < 0.00001	*P* < 0.05	6	Class IV	Moderate
Infertility with polycystic ovary syndrome	Traditional Chinese medicine/(traditional Chinese medicine + acupuncture) + conventional Western medicine	Conventional Western medicine	1	Endometrial spiral artery resistance index	Yang Ting, 2022 (46)	119/237	MD	−0.10 [−0.13,-0.07]	3/3	Fixed	0%; *P* < 0.00001	na	6	Class IV	High
Infertility with polycystic ovary syndrome	Traditional Chinese medicine/(traditional Chinese medicine + acupuncture) + conventional Western medicine	Conventional Western medicine	1	TNF-α	Yang Ting, 2022 (46)	91/181	MD	−1.07 [−1.57,-0.58]	2/2	Fixed	21%; *P* < 0.0001	*P* < 0.05	6	Class IV	Moderate
PCOS infertility	Acupuncture + Western medicine	Western medicine	1	The pregnancy rate	Huang Shouqia, 2021 (37)	568/1071	RR	1.75 [1.50,ss2.03]	14/14	Fixed	0%; *P* < 0.00001	na	6	Class IV	High
PCOS infertility	Acupuncture + Western medicine	Western medicine	1	Ovulation rate during the cycle	Huang Shouqia, 2021 (37)	129/258	RR	1.47 [1.32,1.64]	4/4	Fixed	18%; *P* < 0.00001	na	6	Class IV	High
PCOS infertility	Acupuncture + Western medicine	Western medicine	1	The ovulation rate	Huang Shouqia, 2021 (37)	316/627	RR	1.29 [1.18,1.41]	8/8	Fixed	5%; *P* < 0.00001	na	6	Class IV	High
PCOS infertility	Acupuncture + Western medicine	Western medicine	1	LH	Huang Shouqia, 2021 (37)	287/571	SMD	−1.09[−1.64,−0.53]	7/7	Random	89%; 0.0001	na	6	Class IV	Moderate
PCOS infertility	Acupuncture + Western medicine	Western medicine	1	LH/FSH	Huang Shouqia, 2021 (37)	110/220	SMD	−1.30 [−2.35,−0.25]	3/3	Random	92%; 0.02	na	6	Class IV	Moderate
PCOS infertility	Acupuncture + Western medicine	Western medicine	1	T	Huang Shouqia, 2021 (37)	324/555	SMD	−1.13 [−1.59,−0.66]	8/8	Random	87%; *P* < 0.00001	na	6	Class IV	Moderate
PCOS infertility	Chinese patent medicines/Chinese herbal decoctions + Western medicines	Conventional Western medicine	1	Comprehensive clinical efficacy	Xue Zhu, 2022 (45)	557/1111	OR	3.28 [2.34,4.59]	13/13	Fixed	0%; *P* < 0.00001	*P* > 0.05	7	Class IV	High
PCOS infertility	Chinese patent medicine + Western medicine	Conventional Western medicine	1	Comprehensive clinical efficacy	Xue Zhu, 2022 (45)	125/250	OR	3.91 [1.81,8.44]	3/3	Fixed	0%; 0.0005	*P* > 0.05	6	Class IV	High
PCOS infertility	Chinese herbal decoctions + Western medicine	Conventional Western medicine	1	Comprehensive clinical efficacy	Xue Zhu, 2022 (45)	432/861	OR	3.14 [2.16,4.57]	10/10	Fixed	0%; *P* < 0.00001	*P* > 0.05	6	Class IV	High
PCOS infertility	Chinese patent medicines/Chinese herbal decoctions + Western medicines	Conventional Western medicine	1	The ovulation rate	Xue Zhu, 2022 (45)	427/862	OR	3.07 [2.22,4.25]	7/7	Fixed	48%; *P* < 0.00001	na	6	Class IV	High
PCOS infertility	Chinese patent medicines/Chinese herbal decoctions + Western medicines	Conventional Western medicine	1	The pregnancy rate	Xue Zhu, 2022 (45)	726/1449	OR	3.15 [2.51,3.97]	16/16	Fixed	0%; *P* < 0.00001	*P* > 0.05	7	Class IV	High
PCOS infertility	Chinese patent medicine + Western medicine	Conventional Western medicine	1	The pregnancy rate	Xue Zhu, 2022 (45)	85/132	OR	3.00 [1.85,4.88]	4/4	Fixed	0%; *P* < 0.00001	*P* > 0.05	6	Class IV	High
PCOS infertility	Chinese herbal decoctions + Western medicine	Conventional Western medicine	1	The pregnancy rate	Xue Zhu, 2022 (45)	572/1141	OR	3.20 [2.46,4.15]	12/12	Fixed	0%; *P* < 0.00001	*P* > 0.05	6	Class IV	High
PCOS infertility	Chinese patent medicines/Chinese herbal decoctions + Western medicines	Conventional Western medicine	1	LH	Xue Zhu, 2022 (45)	614/1233	SMD	−0.96 [−1.44,-0.47]	15/15	Random	94%; 0.0001	*P* > 0.05	7	Class IV	Moderate
PCOS infertility	Chinese patent medicines/Chinese herbal decoctions + Western medicines	Conventional Western medicine	1	FSH	Xue Zhu, 2022 (45)	543/1092	SMD	−0.32 [−0.60,-0.04]	13/13	Random	80%; 0.03	*P* > 0.05	7	Class IV	Moderate
PCOS infertility	Chinese patent medicines/Chinese herbal decoctions + Western medicines	Conventional Western medicine	1	LH/FSH	Xue Zhu, 2022 (45)	214/434	SMD	−2.19 [−3.70,-0.68]	6/6	Random	97%; 0.004	na	6	Class IV	Moderate
PCOS infertility	Chinese patent medicines/Chinese herbal decoctions + Western medicines	Conventional Western medicine	1	T	Xue Zhu, 2022 (45)	563/1131	SMD	−1.09 [−1.71,–0.48]	14/14	Random	95%;0.0005	*P* > 0.05	7	Class IV	Moderate
PCOS infertility	Chinese patent medicines/Chinese herbal decoctions + Western medicines	Conventional Western medicine	1	Endometrial thickness	Xue Zhu, 2022 (45)	344/691	MD	1.27 [0.44,2.09]	8/8	Random	94%; 0.003	na	6	Class IV	Moderate
Infertility caused by polycystic ovary syndrome	Reinforcing kidney and activating blood Chinese herbal + Western medicine	Western medicine	1	Total effective rate	Xu Lifang, 2018 (44)	863/1556	OR	3.83 [2.95,4.96]	18/18	Fixed	8%; *P* < 0.00001	*P* < 0.05	4	Class IV	Moderate
Infertility caused by polycystic ovary syndrome	Reinforcing kidney and activating blood Chinese herbal + Western medicine	Western medicine	1	The pregnancy rate	Xu Lifang, 2018 (44)	863/1556	OR	2.83 [2.06,3.88]	18/18	Random	44%; *P* < 0.00001	*P* < 0.05	4	Class IV	Moderate
Infertility caused by polycystic ovary syndrome	Reinforcing kidney and activating blood Chinese herbal + Western medicine	Western medicine	1	Security analysis	Xu Lifang, 2018 (44)	412/731	OR	0.26 [0.09,0.80]	10/10	Random	62%; 0.02	*P* < 0.05	4	Class IV	Low
Infertility in polycystic ovary	Reinforcing kidney and activating blood Chinese herbal + LE	LE	1	Total effective rate	Sheng Nan, 2021 (41)	411/732	OR	4.64 [3.02,7.14]	8/8	Fixed	0%; *P* < 0.00001	*P* < 0.05	7	Class IV	Moderate
Infertility in polycystic ovary	Reinforcing kidney and activating blood Chinese herbal + LE	LE	1	The pregnancy rate	Sheng Nan, 2021 (41)	410/730	OR	3.14 [2.23,4.43]	8/8	Fixed	0%; *P* < 0.00001	*P* < 0.05	6	Class IV	Moderate
Infertility in polycystic ovary	Reinforcing kidney and activating blood Chinese herbal + LE	LE	1	Endometrial thickness	Sheng Nan, 2021 (41)	228/456	SMD	2.48 [1.43,3.52]	6/6	Random	95%; *P* < 0.00001	*P* < 0.05	6	Class IV	Low
Infertility in polycystic ovary	Reinforcing kidney and activating blood Chinese herbal + LE	LE	1	LH	Sheng Nan, 2021 (41)	198/396	SMD	−1.46 [−2.49,-0.43]	5/5	Random	95%; 0.005	*P* < 0.05	6	Class IV	Low
Infertility in polycystic ovary	Reinforcing kidney and activating blood Chinese herbal + LE	LE	1	FSH	Sheng Nan, 2021 (41)	122/244	SMD	−1.07 [−1.34,-0.80]	3/3	Random	43%; *P* < 0.00001	*P* < 0.05	6	Class IV	Moderate
Polycystic ovary syndrome infertility	Bushen Tiaojing recipes + ovulation induction drugs	ovulation induction drugs	1	The ovulation rate	Chen Jing, 2021 (34)	755/1498	RR	1.29 [1.21,1.37]	14/14	Random	0%; *P* < 0.00001	*P* < 0.05	9	Class IV	Moderate
Polycystic ovary syndrome infertility	Bushen Tiaojing recipes + ovulation induction drugs	ovulation induction drugs	1	The pregnancy rate	Chen Jing, 2021 (34)	977/1942	RR	1.67 [1.52,1.84]	19/19	Random	0%; *P* < 0.00001	*P* < 0.05	9	Class IV	Moderate
Polycystic ovary syndrome infertility	Bushen Tiaojing recipes + ovulation induction drugs	ovulation induction drugs	1	Incidence of adverse reactions	Chen Jing, 2021 (34)	252/504	OR	0.44 [0.25,0.79]	5/5	Fixed	0%; 0.006	na	8	Class IV	High
Polycystic ovary syndrome infertility	Bushen Tiaojing recipes + ovulation induction drugs	ovulation induction drugs	1	T	Chen Jing, 2021 (34)	712/1430	MD	−0.22 [−0.34,−0.11]	16/16	Random	94%; *P* < 0.0001	na	8	Class IV	Moderate
Polycystic ovary syndrome infertility	Bushen Tiaojing recipes + ovulation induction drugs	ovulation induction drugs	1	LH	Chen Jing, 2021 (34)	741/1486	MD	−2.23 [−2.71,−1.76]	17/17	Random	86%; *p* < 0.00001	na	8	Class IV	Moderate
Polycystic ovary syndrome infertility	Bushen Tiaojing recipes + ovulation induction drugs	ovulation induction drugs	1	Endometrial thickness	Chen Jing, 2021 (34)	647/1291	MD	1.38 [0.88,1.89]	13/13	Random	95%; *P* < 0.00001	na	8	Class IV	Moderate
Polycystic ovary syndrome infertility	Bushen Tiaojing recipes + ovulation induction drugs	ovulation induction drugs	1	Ovarian volume	Chen Jing, 2021 (34)	200/397	MD	−2.00 [−2.33,−1.67]	5/5	Random	68%; *P* < 0.00001	na	8	Class IV	Moderate
Infertility with PCOS	Dingkundan + LE	LE	1	Endometrial thickness	DING Ke-fan, 2024 (35)	459/918	MD	1.71 [1.32,2.11]	8/8	Random	79%; *P* < 0.00001	*P* > 0.05	7	Class IV	Moderate
Infertility with PCOS	Dingkundan + LE	LE	1	The ovulation rate	DING Ke-fan, 2024 (35)	328/591	RR	1.25 [1.16,1.34]	6/6	Fixed	68%; *P* < 0.00001	*P* < 0.05	7	Class IV	Low
Infertility with PCOS	Dingkundan + LE	LE	1	The pregnancy rate	DING Ke-fan, 2024 (35)	509/1018	RR	1.62 [1.44,1.84]	9/9	Fixed	12%; *P* < 0.00001	*P* < 0.05	7	Class IV	Moderate
Infertility with PCOS	Dingkundan + other Western medicines	Other Western medicines	1	Endometrial thickness	DING Ke-fan, 2024 (35)	206/404	MD	2.36 [1.13,3.59]	4/4	Random	92%; 0.002	*P* > 0.05	7	Class IV	Moderate
Infertility with PCOS	Dingkundan + other Western medicines	Other Western medicines	1	The ovulation rate	DING Ke-fan, 2024 (35)	309/553	RR	1.26 [1.17,1.36]	6/6	Fixed	0%; P < 0.00001	*P* < 0.05	7	Class IV	Moderate
Infertility with PCOS	Dingkundan + other Western medicines	Other Western medicines	1	The pregnancy rate	DING Ke-fan, 2024 (35)	344/681	RR	1.55 [1.33,1.80]	7/7	Fixed	30%; *P* < 0.00001	*P* < 0.05	7	Class IV	Moderate
Infertility with PCOS	Dingkundan + Western medicine	Western medicine	1	Endometrial thickness	DING Ke-fan, 2024 (35)	665/1322	MD	1.90 [1.48,2.32]	12/12	Random	85%; P < 0.00001	*P* > 0.05	6	Class IV	Moderate
Infertility with PCOS	Dingkundan + Western medicine	Western medicine	1	The number of mature follicles	DING Ke-fan, 2024 (35)	564/1121	MD	0.81 [0.57,1.06]	10/10	Random	93%; *P* < 0.00001	*P* > 0.05	7	Class IV	Moderate
Infertility with PCOS	Dingkundan + Western medicine	Western medicine	1	The ovulation rate	DING Ke-fan, 2024 (35)	717/1432	RR	1.25 [1.19,1.32]	12/12	Fixed	43%; *P* < 0.00001	*P* < 0.05	6	Class IV	Moderate
Infertility with PCOS	Dingkundan + Western medicine	Western medicine	1	The pregnancy rate	DING Ke-fan 2024 (35)	853/1699	RR	1.59 [1.45,1.75]	16/16	Fixed	17%; *P* < 0.00001	*P* < 0.05	6	Class IV	Moderate
Infertility with PCOS	Dingkundan + Western medicine	Western medicine	1	LH	DING Ke-fan 2024 (35)	757/1512	MD	−2.21 [−2.77,-1.65]	12/12	Random	94%; *P* < 0.00001	*P* < 0.05	7	Class IV	Low
Infertility with PCOS	Dingkundan + Western medicine	Western medicine	1	Total effective rate	DING Ke-fan 2024 (35)	256/512	RR	1.29 [1.19,1.40]	6/6	Fixed	8%; *P* < 0.00001	*P* < 0.05	7	Class IV	Moderate
Infertility in patients with polycystic ovary syndrome	Jinfeng pills + Western medicine	Western medicine	1	The effective rates	Yu Xu, 2022 (32)	302/592	RR	1.15 [1.03,1.28]	6/6	Fixed	0%; 0.989	*P* > 0.05	8	Class IV	High
Infertility in patients with polycystic ovary syndrome	Jinfeng pills + Western medicine	Western medicine	1	FSH	Yu Xu, 2022 (32)	224/415	MD	−5.10 [−7.95,–2.24]	4/4	Random	98%; 0.0001	*P* > 0.05	8	Class IV	Moderate
Infertility in patients with polycystic ovary syndrome	Jinfeng pills + Western medicine	Western medicine	1	E2	Yu Xu, 2022 (32)	201/369	MD	10.74 [4.19,17.29]	4/4	Random	85%; 0.0002	*P* > 0.05	8	Class IV	Moderate
Infertility in patients with polycystic ovary syndrome	Jinfeng pills + Western medicine	Western medicine	1	T	Yu Xu, 2022 (32)	212/412	MD	−1.17 [−2.09,–0.25]	4/4	Random	99%; *P* < 0.00001	*P* > 0.05	8	Class IV	Moderate
Infertility in patients with polycystic ovary syndrome	Jinfeng pills + Western medicine	Western medicine	1	The pregnancy rates	Yu Xu, 2022 (32)	138/276	RR	1.36 [1.13,1.64]	3/3	Fixed	0%;0.908	*P* > 0.05	8	Class IV	High
Polycystic ovary syndrome infertility	Kuntai capsules + CC	CC	1	The pregnancy rates	Liu Ying, 2019 (38)	139/278	RR	1.68 [1.15,2.44]	2/2	Fixed	0%; 0.007	*P* < 0.05	5	Class IV	Moderate
Polycystic ovary syndrome infertility	Kuntai capsules + ethinylestradiol and cyproterone acetate tablets + CC + HMG	Ethinylestradiol and cyproterone acetate tablets + CC + HMG	1	The pregnancy rates	Liu Ying, 2019 (38)	74/148	RR	1.50 [1.08,2.09]	2/2	Fixed	0%, 0.02	*P* < 0.05	5	Class IV	Moderate
Polycystic ovary syndrome infertility	Kuntai capsules + ethinylestradiol and cyproterone acetate tablets + CC + HMG	Ethinylestradiol and cyproterone acetate tablets + CC + HMG	1	The pregnancy rates	Liu Ying, 2019 (38)	74/148	RR	1.42 [1.14,1.77]	2/2	Fixed	0%, 0.002	*P* < 0.05	5	Class IV	Moderate
Polycystic ovary syndrome infertility	Kuntai capsules + LE	LE	1	The pregnancy rates	Liu Ying, 2019 (38)	176/350	RR	2.15 [1.48,3.14]	3/3	Fixed	0%; *P* < 0.0001	*P* < 0.05	5	Class IV	Moderate
Polycystic ovary syndrome infertility	Kuntai capsules + LE	LE	1	The pregnancy rates	Liu Ying, 2019 (38)	42/84	RR	1.31 [1.05,1.64]	1/1	Fixed	na; 0.02	*P* < 0.05	5	Class IV	Moderate
Polycystic ovary syndrome infertility	Kuntai capsules + ethinylestradiol and cyproterone acetate tablets	Ethinylestradiol and cyproterone acetate tablets	1	The pregnancy rates	Liu Ying, 2019 (38)	78/156	RR	1.44 [1.21,1.72]	2/2	Fixed	0%; *P* < 0.0001	*P* < 0.05	5	Class IV	Moderate
Polycystic ovary syndrome infertility	Kuntai capsules + Western medicine	Western medicine	1	The pregnancy rates	Liu Ying, 2019 (38)	564/1126	RR	1.71 [1.46,2.01]	11/11	Fixed	8%; *P* < 0.00001	*P* < 0.05	5	Class IV	Moderate
Polycystic ovary syndrome infertility	Kuntai capsules + Western medicine	Western medicine	1	The pregnancy rates	Liu Ying, 2019 (38)	386/772	RR	1.34 [1.23,1.46]	8/8	Fixed	9%; *P* < 0.00001	*P* < 0.05	5	Class IV	Moderate
Infertility with PCOS	A combination of IVF-ET and CHM	IVF-ET + blank/placebo	1	The clinical pregnancy rate	Chang Liu, 2021 (30)	336/663	OR	2.41 [1.73,3.35]	10/10	Fixed	0%; *P* < 0.00001	*P* < 0.05	8	Class IV	Moderate
Infertility with PCOS	A combination of IVF-ET and CHM	IVF-ET + blank/placebo	1	The OHSS incidence.	Chang Liu, 2021 (30)	305/599	OR	0.31 [0.18,0.55]	9/9	Fixed	0%; *P* < 0.0001	*P* < 0.05	7	Class IV	Moderate
Infertility with PCOS	A combination of IVF-ET and CHM (Decoction)	IVF-ET + blank/placebo	1	The clinical pregnancy rate	Chang Liu, 2021 (30)	212/421	OR	2.36 [1.56,3.57]	6/6	Fixed	0%; *P* < 0.0001	*P* < 0.05	7	Class IV	Moderate
Infertility with PCOS	A combination of IVF-ET and CHM (Granules)	IVF-ET + blank/placebo	1	The clinical pregnancy rate	Chang Liu, 2021 (30)	97/196	OR	2.89 [1.56,5.34]	3/3	Fixed	0%; 0.0007	*P* < 0.05	7	Class IV	Moderate
Infertility with PCOS	A combination of IVF-ET and CHM (Decoction + Granules)	IVF-ET + blank/placebo	1	The clinical pregnancy rate	Chang Liu, 2021 (30)	309/617	OR	2.51 [1.78,3.54]	9/9	Fixed	0%; *P* < 0.00001	*P* < 0.05	7	Class IV	Moderate
Infertility with PCOS	A combination of IVF-ET and CHM (before oocyte extraction)	IVF-ET + blank/placebo	1	The clinical pregnancy rate	Chang Liu, 2021 (30)	244/480	OR	2.36 [1.59,3.49]	7/7	Fixed	0%; *P* < 0.0001	*P* < 0.05	7	Class IV	Moderate
Infertility with PCOS	A combination of IVF-ET (fresh embryo) and CHM	IVF-ET (fresh embryo) + blank/placebo	1	The clinical pregnancy rate	Chang Liu, 2021 (30)	274/548	OR	2.67 [1.85,3.87]	8/8	Fixed	0%; *P* < 0.00001	*P* < 0.05	7	Class IV	Moderate
Infertility with PCOS	A combination of IVF-ET (frozen and fresh embryo) and CHM	IVF-ET (frozen and fresh embryo) + blank/placebo	1	the clinical pregnancy rate	Chang Liu, 2021 (30)	336/671	OR	2.47 [1.78,3.44]	10/10	Fixed	0%; *P* < 0.00001	*P* < 0.05	7	Class IV	Moderate
Infertility with PCOS	A combination of IVF-ET (fresh embryo, 2–3 days transfer) and CHM	IVF-ET (fresh embryo, 2–3 days transfer) + blank/placebo	1	the clinical pregnancy rate	Chang Liu, 2021 (30)	118/238	OR	2.73 [1.59,4.69]	4/4	Fixed	0%; 0.0003	*P* < 0.05	7	Class IV	Moderate
Infertility with PCOS	A combination of IVF-ET (fresh embryo, 18–22 h transfer) and CHM	IVF-ET (fresh embryo, 18–22 h transfer) + blank/placebo	1	the clinical pregnancy rate	Chang Liu, 2021 (30)	86/172	OR	2.67 [1.31,5.43]	2/2	Fixed	0%; 0.007	*P* < 0.05	7	Class IV	Moderate
Infertility with PCOS	A combination of IVF-ET (fresh embryo, 2–3 days transfer and fresh embryo, 18–22 h transfer) and CHM	IVF-ET (fresh embryo, 2–3 days transfer and fresh embryo, 18–22 h transfer) + blank/placebo	1	the clinical pregnancy rate	Chang Liu, 2021 (30)	204/410	OR	2.71 [1.76,4.16]	6/6	Fixed	0%; *P* < 0.00001	*P* < 0.05	7	Class IV	Moderate
PCOS infertility	Combine traditional Chinese and Western medicine	Western medicine	3	The ovulation rate	Cai Shu, 2016 (29)	716/1355	POR	2.14 [1.67,2.73]	11/11	Fixed	58%; *P* < 0.00001	*P* > 0.05	5	Class IV	Moderate
PCOS infertility	Combine traditional Chinese and Western medicine	Western medicine	3	The pregnancy rate	Cai Shu, 2016 (29)	530/1069	POR	3.28 [2.56,4.22]	13/13	Fixed	0%; *P* < 0.00001	*P* > 0.05	5	Class IV	High
PCOS infertility	Combine traditional Chinese and Western medicine	Western medicine	3	Effective rate	Cai Shu, 2016 (29)	227/449	POR	3.45 [2.09,5.69]	6/6	Fixed	0%; *P* < 0.00001	*P* > 0.05	5	Class IV	High
Infertility with PCOS	Chinese medicine + Western medicine (Traditional Chinese Medicine Cycle Method)	Western medicine	1	Total clinical efficiency	Wu Haiyan, 2019 (42)	2571/5126	OR	3.78 [3.29,4.34]	58/58	Fixed	25%; *P* < 0.00001	*P* < 0.05	6	Class II	Moderate
Infertility with PCOS	Chinese medicine + Western medicine (Traditional Chinese Medicine Cycle Method)	Western medicine	1	The ovulation rate	Wu Haiyan, 2019 (42)	1115/2228	OR	2.90 [2.39,3.50]	26/26	Fixed	55%; *P* < 0.00001	na	5	Class II	Moderate
Infertility with PCOS	Chinese medicine + Western medicine (Traditional Chinese Medicine Cycle Method)	Western medicine	1	The pregnancy rate	Wu Haiyan, 2019 (42)	2193/4350	OR	2.76 [2.43,3.13]	51/51	Fixed	0%; *P* < 0.00001	na	5	Class I	High
Infertility with PCOS	Acupuncture + Western medicine (Traditional Chinese Medicine Cycle Method)	Western medicine	1	Total clinical efficiency	Wu Haiyan, 2019 (42)	918/1814	OR	2.64 [2.14,3.25]	19/19	Fixed	11%; *P* < 0.00001	*P* < 0.05	6	Class IV	Moderate
Infertility with PCOS	Acupuncture + Western medicine (Traditional Chinese Medicine Cycle Method)	Western medicine	1	The ovulation rate	Wu Haiyan, 2019 (42)	521/1032	OR	2.74 [2.04,3.68]	11/11	Fixed	0%; *P* < 0.00001	na	5	Class IV	High
Infertility with PCOS	Acupuncture + Western medicine (Traditional Chinese Medicine Cycle Method)	Western medicine	1	The pregnancy rate	Wu Haiyan, 2019 (42)	803/1582	OR	2.45 [1.98,3.04]	16/16	Fixed	0%; *P* < 0.00001	na	5	Class IV	High
Infertility caused by polycystic ovary syndrome	Traditional Chinese medicine cycle therapy + ovulation induction protocol	Ovulation induction protocol	2	The pregnancy rate	Li Huimin, 2024 (39)	594/1171	RR	1.80 [1.58,2.04]	15/15	Fixed	0%; *P* < 0.00001	*P* > 0.05	8	Class IV	High
Infertility caused by polycystic ovary syndrome	Traditional Chinese medicine cycle therapy + ovulation induction protocol	Ovulation induction protocol	2	Total effective rate	Li Huimin, 2024 (39)	278/554	RR	1.25 [1.15,1.37]	8/8	Fixed	0%; *P* < 0.00001	na	7	Class IV	High
Infertility caused by polycystic ovary syndrome	Traditional Chinese medicine cycle therapy + ovulation induction protocol	Ovulation induction protocol	2	The ovulation rate	Li Huimin, 2024 (39)	1150/2301	RR	1.10 [1.06,1.14]	11/11	Fixed	18%; *P* < 0.00001	na	7	Class III	High
Infertility caused by polycystic ovary syndrome	Traditional Chinese medicine cycle therapy + ovulation induction protocol	Ovulation induction protocol	1	Androgen level	Li Huimin, 2024 (39)	156/298	MD	−0.39 [−0.52,-0.26]	6/6	Fixed	0%; *P* < 0.00001	na	7	Class IV	High
Infertility caused by polycystic ovary syndrome	Traditional Chinese medicine cycle therapy + ovulation induction protocol	Ovulation induction protocol	1	LH	Li Huimin, 2024 (39)	185/357	MD	−1.49 [−1.93,-1.05]	6/6	Fixed	0%; *P* < 0.00001	na	7	Class IV	High
Infertility caused by polycystic ovary syndrome	Traditional Chinese medicine cycle therapy + ovulation induction protocol	Ovulation induction protocol	2	Endometrial thickness	Li Huimin, 2024 (39)	86/171	MD	1.12 [0.94,1.29]	3/3	Fixed	42%; *P* < 0.00001	na	7	Class IV	High
Infertility caused by polycystic ovary syndrome	Traditional Chinese medicine cycle therapy + ovulation induction protocol	Ovulation induction protocol	1	Incidence of OHSS	Li Huimin, 2024 (39)	112/219	RR	0.25 [0.08,0.79]	3/3	Fixed	0%; 0.02	na	7	Class IV	High
Infertility caused by polycystic ovary syndrome	Traditional Chinese medicine cycle therapy + ovulation induction protocol	Ovulation induction protocol	1	Adverse reaction rate	Li Huimin, 2024 (39)	288/573	RR	0.36 [0.19,0.67]	7/7	Fixed	6%; 0.001	na	7	Class IV	High
Non-significant															
Infertility with polycystic ovary syndrome	Compound Xuanju capsules + CC	CC	1	The pregnancy rate	Zhou Kunyan, 2014 (48)	95/190	RR	1.52 [0.61,2.27]	3/3	Fixed	0%; 0.04	*P* < 0.05	5	NS	Moderate
Infertility with polycystic ovary syndrome	Compound Xuanju capsules + CC	CC	1	Ovulation rate during the cycle	Zhou Kunyan, 2014 (48)	80/162	RR	1.09 [0.86,1.36]	1/1	Fixed	na; 0.48	*P* < 0.05	5	NS	Moderate
Infertility with polycystic ovary syndrome	Compound Xuanju capsules + LE	LE	1	The pregnancy rate	Zhou Kunyan, 2014 (48)	45/90	RR	1.69 [0.98,2.92]	1/1	Fixed	na; 0.06	*P* < 0.05	5	NS	Moderate
Infertility caused by polycystic ovary syndrome	Traditional Chinese medicine for tonifying the kidney and enhancing Yang + Western medicine for inducing ovulation	Western medicine for inducing ovulation	1	Follicle-stimulating hormone	Zhao Shuai, 2022 (47)	364/726	SMD	0.21 [−0.27,0.68]	10/10	Random	90%; 0.40	na	6	NS	Moderate
Infertility caused by polycystic ovary syndrome	Traditional Chinese medicine for tonifying the kidney and enhancing Yang + Western medicine for inducing ovulation	Western medicine for inducing ovulation	1	Estradiol	Zhao Shuai, 2022 (47)	301/600	SMD	−1.63 [−3.53,0.27]	8/8	Random	98%; 0.73	na	6	NS	Moderate
PCOS infertility	Chinese patent medicines/Chinese herbal decoctions + Western medicines	Western medicine	1	The rate of spontaneous abortion	Xue Zhu, 2022 (45)	119/224	OR	0.82 [0.28,2.39]	2/2	Fixed	0%; 0.71	na	6	NS	High
Polycystic ovary syndrome infertility	Bushen Tiaojing recipes + ovulation induction drugs	ovulation induction drugs	1	Estradiol	Chen Jing, 2021 (34)	687/1378	MD	1.84 [−3.40,7.09]	15/15	Random	88%; 0.49	na	8	NS	Moderate
Polycystic ovary syndrome infertility	Bushen Tiaojing recipes + ovulation induction drugs	ovulation induction drugs	1	FSH	Chen Jing, 2021 (34)	709/1422	MD	−0.15 [−0.47,0.17]	16/16	Random	90%; 0.36	na	8	NS	Moderate
Infertility in patients with polycystic ovary syndrome	Jinfeng pills + Western medicine	Western medicine	1	LH	Yu Xu, 2022 (32)	224/415	MD	−0.37 [−3.71,2.97]	4/4	Random	98%; 0.0001	*P* > 0.05	8	NS	Moderate
Infertility in patients with polycystic ovary syndrome	Jinfeng pills + Western medicine	Western medicine	1	The ovulation rates	Yu Xu, 2022 (32)	138/276	RR	1.09 [0.91,1.30]	3/3	Fixed	0%; 0.805	*P* > 0.05	8	NS	High
LIU polycystic ovary syndrome infertility	Kuntai capsules + CC	CC	1	The ovulation rate	Liu Ying, 2019 (38)	139/278	RR	1.28 [0.95,1.72]	2/2	Random	78%; 0.11	*P* < 0.05	5	NS	Low
Polycystic ovary syndrome infertility	Kuntai capsules + CC + HCG	CC + HCG	1	The pregnancy rate	Liu Ying, 2019 (38)	53/106	RR	1.50 [0.86,2.62]	1/1	Random	na; 0.16	*P* < 0.05	5	NS	Moderate
Polycystic ovary syndrome infertility	Kuntai capsules + CC + HCG	CC + HCG	1	The ovulation rate	Liu Ying, 2019 (38)	53/106	RR	1.17 [0.92,1.49]	1/1	Random	na; 0.20	*P* < 0.05	5	NS	Moderate
Polycystic ovary syndrome infertility	Kuntai capsules + ethinylestradiol and cyproterone acetate tablets	Ethinylestradiol and cyproterone acetate tablets	1	The pregnancy rate	Liu Ying, 2019 (38)	122/244	RR	1.83 [0.98,3.43]	3/3	Random	66%; 0.06	*P* < 0.05	5	NS	Low
Chang Liu, 2021 (30)	Combination of IVF-ET and CHM	IVF-ET + blank/placebo	1	The abortion rate	Chang Liu, 2021 (30)	77/125	OR	0.64 [0.23,1.81]	4/4	Fixed	0%; 0.4	*P* < 0.05	7	NS	Moderate
Infertility with PCOS	A combination of IVF-ET and CHM (over all the process of IVF)	IVF-ET + blank/placebo	1	The clinical pregnancy rate	Chang Liu, 2021 (30)	62/123	OR	1.81 [0.86,3.80]	2/2	Fixed	0%; 0.12	*P* < 0.05	7	NS	Moderate
Infertility with PCOS	A combination of IVF-ET (frozen embryo) and CHM	IVF-ET (frozen embryo) + blank/placebo	1	The clinical pregnancy rate	Chang Liu, 2021 (30)	62/123	OR	1.81 [0.86,3.80]	2/2	Fixed	0%; 0.12	*P* < 0.05	7	NS	Moderate
PCOS infertility	Acupuncture + Western medicine	Western medicine	1	FSH	Huang Shouqia, 2021 (37)	287/571	SMD	0.31 [0.00,0.61]	7/7	Random	69%; 0.05	na	6	Class IV	Moderate

CC: clomiphene citrate; LE: letrozole; HCG: human chorionic gonadotropin; HMG: urogonadotropin; CHM: Chinese herbal medicine; IVF-ET: in vitro fertilization-embryo transfer; T: testosterone; BMI: body mass index; LH: luteinizing hormone; FSH: follicle-stimulating hormone; E2: estradiol; BBT: basal body temperature; HGF: hepatocyte growth factor; VEGF: vascular endothelial growth factor; TNF-α: tumor necrosis factor-α; OHSS: ovarian hyper-stimulation syndrome; MD: mean difference; SMD: standardized mean difference; OR: odds ratio; RR: risk ratio; POR: Peto odds ratio; NS: non-significant; T: total; R: RCT.

### Significant effects

#### Reproductive outcomes

Integrated traditional Chinese and Western medicine interventions showed significant benefits for several reproductive outcomes in women with PCOS complicated by infertility. Compared with Western medicine or ovulation-induction drugs alone, Compound Xuanju capsules combined with clomiphene, letrozole, hormones, or other Western medicines significantly increased pregnancy and ovulation-related outcomes. Compound Xuanju capsules plus hormones increased ovulation rate (OR: 2.04, 95% CI: 1.46, 2.84) and pregnancy rate (OR: 2.43, 95% CI: 1.84, 3.21), while compound Xuanju capsules plus Western medicine improved pregnancy rate (RR: 1.68, 95% CI: 1.45, 1.94) and cycle ovulation rate (RR: 1.17, 95% CI: 1.03, 1.34).

Kidney-tonifying and yang-invigorating Chinese herbal therapies combined with Western ovulation-induction drugs increased pregnancy rate (RR: 1.95, 95% CI: 1.66, 2.28). Chinese medicine alone or combined with acupuncture plus conventional Western medicine also increased ovulation rate (OR: 2.42, 95% CI: 1.89, 3.11) and pregnancy rate (OR: 3.10, 95% CI: 2.65, 3.63) and reduced miscarriage rate (OR: 0.33, 95% CI: 0.21, 0.53). Acupuncture plus Western medicine increased pregnancy rate (RR: 1.75, 95% CI: 1.50, 2.03), cycle ovulation rate (RR: 1.47, 95% CI: 1.32, 1.64), and ovulation rate (RR: 1.29, 95% CI: 1.18, 1.41).

Chinese patent medicines or Chinese herbal decoctions combined with Western medicine increased ovulation rate (OR: 3.07, 95% CI: 2.22, 4.25) and pregnancy rate, including Chinese patent medicines or decoctions plus Western medicine (OR: 3.15, 95% CI: 2.51, 3.97), Chinese patent medicines plus Western medicine (OR: 3.00, 95% CI: 1.85, 4.88), and Chinese herbal decoctions plus Western medicine (OR: 3.20, 95% CI: 2.46, 4.15). Kidney-tonifying and blood-activating Chinese herbal therapies plus Western medicine increased pregnancy rate (OR: 2.83, 95% CI: 2.06, 3.88), while the same therapies plus letrozole increased pregnancy rate (OR: 3.14, 95% CI: 2.23, 4.43). Kidney-tonifying and menstruation-regulating formulas plus ovulation-inducing drugs increased ovulation rate (RR: 1.29, 95% CI: 1.21, 1.37) and pregnancy rate (RR: 1.67, 95% CI: 1.52, 1.84).

Dingkun Dan combined with letrozole, other Western medicines, or Western medicine significantly improved ovulation rates compared with letrozole (RR: 1.25, 95% CI: 1.16, 1.34), other Western medicines (RR: 1.26, 95% CI: 1.17, 1.36), and Western medicine overall (RR: 1.25, 95% CI: 1.19, 1.32). It also increased pregnancy rates compared with letrozole (RR: 1.62, 95% CI: 1.44, 1.84), other Western medicines (RR: 1.55, 95% CI: 1.33, 1.80), and Western medicine overall (RR: 1.59, 95% CI: 1.45, 1.75). Jinfeng Wan plus Western medicine increased pregnancy rate (RR: 1.36, 95% CI: 1.13, 1.64). Kuntai capsules combined with clomiphene, ethinylestradiol–cyproterone acetate plus clomiphene plus HMG, letrozole, or Western medicine improved pregnancy rates and ovulation rates compared with corresponding Western medicine controls.

In assisted reproductive technology settings, IVF-ET combined with Chinese herbal medicine significantly increased clinical pregnancy rate (OR: 2.41, 95% CI: 1.73, 3.35). Significant improvements were observed across different Chinese herbal medicine formulations and treatment timings, including decoctions (OR: 2.36, 95% CI: 1.56, 3.57), granules (OR: 2.89, 95% CI: 1.56, 5.34), combined decoction and granules (OR: 2.51, 95% CI: 1.78, 3.54), administration before oocyte retrieval (OR: 2.36, 95% CI: 1.59, 3.49), during fresh embryo transfer (OR: 2.67, 95% CI: 1.85, 3.87), during frozen or mixed embryo transfer cycles (OR: 2.47, 95% CI: 1.78, 3.44), at fresh embryo day 2–3 transfer (OR: 2.73, 95% CI: 1.59, 4.69), at fresh embryo 18–22 h transfer (OR: 2.67, 95% CI: 1.31, 5.43), and during combined fresh embryo transfer windows (OR: 2.71, 95% CI: 1.76, 4.16).

#### Clinical effectiveness and menstrual outcomes

Several integrated interventions improved overall clinical effectiveness, TCM syndrome outcomes, and menstrual-related outcomes. Compound Xuanju capsules plus clomiphene or letrozole significantly improved menstrual improvement rates compared with clomiphene (RR: 1.67, 95% CI: 1.30, 2.14) and letrozole (RR: 2.00, 95% CI: 1.36, 2.94). Compound Xuanju capsules plus hormones improved overall effective treatment rate (OR: 5.35, 95% CI: 3.22, 8.89), total TCM effective rate (OR: 4.73, 95% CI: 2.00, 11.19), and basal body temperature (BBT) improvement (OR: 2.65, 95% CI: 1.53, 4.57). Compound Xuanju capsules plus Western medicine improved TCM syndrome outcomes (RR: 1.60, 95% CI: 1.28, 2.00).

Kidney-tonifying and yang-invigorating Chinese herbal therapies plus Western ovulation-induction drugs increased the total effective rate (RR: 1.32, 95% CI: 1.19, 1.46). Chinese medicine alone or combined with acupuncture plus conventional Western medicine improved overall clinical efficacy (OR: 3.18, 95% CI: 2.37, 4.27). Chinese patent medicines or decoctions plus Western medicine improved the overall clinical effective rate, including Chinese patent medicines or decoctions plus Western medicine (OR: 3.28, 95% CI: 2.34, 4.59), Chinese patent medicines plus Western medicine (OR: 3.91, 95% CI: 1.81, 8.44), and Chinese herbal decoctions plus Western medicine (OR: 3.14, 95% CI: 2.16, 4.57).

Kidney-tonifying and blood-activating Chinese herbal therapies plus Western medicine increased total effective rate (OR: 3.83, 95% CI: 2.95, 4.96), and the same therapies plus letrozole also increased total effective rate (OR: 4.64, 95% CI: 3.02, 7.14). Kidney-tonifying and menstruation-regulating formulas plus ovulation-inducing drugs, Dingkun Dan plus Western medicine, Jinfeng Wan plus Western medicine, integrated traditional Chinese, and Western medicine, TCM cycle-regulation therapy plus Western medicine, acupuncture-based TCM cycle therapy plus Western medicine, and TCM periodic therapy plus ovulation-induction protocols all showed significant improvements in overall effectiveness.

#### Endocrine and hormonal outcomes

Integrated interventions were associated with significant improvements in endocrine and hormonal indicators. Compound Xuanju capsules plus clomiphene reduced serum testosterone compared with clomiphene alone (MD: –0.34, 95% CI: −0.40, −0.28). Compound Xuanju capsules plus hormones increased follicle-stimulating hormone (FSH) (MD: 0.91, 95% CI: 0.32, 1.49) and estradiol (E2) (MD: 15.78, 95% CI: 7.96, 23.60), while reducing luteinizing hormone (LH) (MD: –2.47, 95% CI: −3.07, −1.86), testosterone (MD: –0.36, 95% CI: −0.52, −0.20), and the LH/FSH ratio (MD: -0.45, 95% CI: −0.67, −0.22). Compound Xuanju capsules plus Western medicine reduced testosterone levels (MD: -0.53, 95% CI: −0.90, −0.16).

Kidney-tonifying and yang-invigorating Chinese herbal therapies plus Western ovulation-induction drugs reduced LH (SMD: -1.39, 95% CI: −1.78, −0.99), testosterone (SMD: -1.34, 95% CI: −1.93, −0.75), and LH/FSH ratio (SMD: -1.73, 95% CI: −2.18, −1.29). Acupuncture plus Western medicine reduced LH (SMD: -1.09, 95% CI: −1.64, −0.53), LH/FSH ratio (SMD: -1.30, 95% CI: −2.35, −0.25), and testosterone (SMD: -1.13, 95% CI: −1.59, −0.66). Chinese patent medicines or herbal decoctions plus Western medicine reduced LH (SMD: -0.96, 95% CI: −1.44, −0.47), LH/FSH ratio (SMD: –2.19, 95% CI: −3.70, −0.68), and testosterone (SMD: -1.09, 95% CI: −1.71, −0.48).

Kidney-tonifying and blood-activating therapies plus letrozole reduced LH (SMD: -1.46, 95% CI: −2.49, −0.43) and FSH (SMD: –1.07, 95% CI: −1.34, −0.80). Kidney-tonifying and menstruation-regulating formulas plus ovulation-inducing drugs reduced testosterone (MD: –0.22, 95% CI: −0.34, −0.11) and LH (MD: –2.23, 95% CI: −2.71, −1.76). Dingkun Dan plus Western medicine reduced LH levels (MD: –2.21, 95% CI: −2.77, −1.65), while Jinfeng Wan plus Western medicine increased E2 levels (MD: 10.74, 95% CI: 4.19, 17.29) and reduced FSH (MD: –5.10, 95% CI: −7.95, −2.24) and testosterone levels (MD: -1.17, 95% CI: −2.09, −0.25). TCM periodic therapy plus ovulation-induction protocols also reduced androgen levels (MD: –0.39, 95% CI: −0.52, −0.26) and LH levels (MD: –1.49, 95% CI: −1.93, −1.05).

#### Endometrial receptivity, ovarian morphology, and follicular development

Several interventions improved endometrial receptivity, ovarian morphology, and follicular development. Compound Xuanju capsules plus hormones increased maximum follicle diameter (MD: 2.52, 95% CI: 0.68, 4.37) and endometrial thickness (MD: 1.60, 95% CI: 0.69, 2.51), while reducing left antral follicle count (MD: -1.13, 95% CI: −1.85, −0.42), right antral follicle count (MD: –1.36, 95% CI: −2.00, −0.72), total follicle count (MD: –1.23, 95% CI: −1.56, −0.89), left ovarian volume (MD: -0.96, 95% CI: −1.20, −0.72), and right ovarian volume (MD: –1.09, 95% CI: −1.34, −0.85). Compound Xuanju capsules plus Western medicine increased endometrial thickness (MD: 1.56, 95% CI: 1.26, 1.86).

Kidney-tonifying and yang-invigorating Chinese herbal therapies plus Western ovulation-induction drugs increased endometrial thickness (SMD: 0.97, 95% CI: 0.80, 1.15) and reduced endometrial spiral artery resistance index (MD: -0.23, 95% CI: −0.28, −0.19) and pulsatility index (SMD: –1.16, 95% CI: −1.51, −0.82). Chinese medicine alone or combined with acupuncture plus conventional Western medicine increased endometrial thickness (MD: 1.81, 95% CI: 1.52, 2.10) and reduced endometrial spiral artery pulsatility index (MD: -0.23, 95% CI: −0.29, −0.16) and resistance index (MD: -0.10, 95% CI: −0.13, −0.07).

Chinese patent medicines or herbal decoctions plus Western medicine increased endometrial thickness (MD: 1.27, 95% CI: 0.44, 2.09). Kidney-tonifying and blood-activating therapies plus letrozole increased endometrial thickness (SMD: 2.48, 95% CI: 1.43, 3.52). Kidney-tonifying and menstruation-regulating formulas plus ovulation-inducing drugs increased endometrial thickness (MD: 1.38, 95% CI: 0.88, 1.89) and reduced ovarian volume (MD: –2.00, 95% CI: −2.33, −1.67). Dingkun Dan combined with letrozole, other Western medicines, or Western medicine increased endometrial thickness and mature follicle number (MD: 0.81, 95% CI: 0.57, 1.06). TCM periodic therapy combined with ovulation-induction protocols also increased endometrial thickness (MD: 1.12, 95% CI: 0.94, 1.29).

#### Metabolic, inflammatory, angiogenic, and safety outcomes

Some interventions also improved metabolic, inflammatory, angiogenic, and safety-related outcomes. Compound Xuanju capsules plus letrozole reduced body mass index (BMI) compared with letrozole alone (MD: -3.12, 95% CI: −3.55, −2.69). Compound Xuanju capsules plus hormones reduced hepatocyte growth factor (HGF) (MD: –85.40, 95% CI: −104.97, −65.82) and vascular endothelial growth factor (VEGF) (MD: –18.46, 95% CI: −22.43, −14.49). Chinese medicine alone or combined with acupuncture plus conventional Western medicine reduced serum tumor necrosis factor-alpha (TNF-*α*) (MD: –1.07, 95% CI: −1.57, −0.58).

Regarding safety outcomes, Chinese medicine alone or combined with acupuncture plus conventional Western medicine reduced adverse event incidence (OR: 0.53, 95% CI: 0.36, 0.80). Kidney-tonifying and blood-activating Chinese herbal therapies plus Western medicine reduced adverse events (OR: 0.26, 95% CI: 0.09, 0.80). Kidney-tonifying and menstruation-regulating formulas plus ovulation-inducing drugs reduced adverse events (OR: 0.44, 95% CI: 0.25, 0.79). In assisted reproductive technology settings, IVF-ET combined with Chinese herbal medicine reduced ovarian hyperstimulation syndrome incidence (OR: 0.31, 95% CI: 0.18, 0.55). TCM periodic therapy combined with ovulation-induction protocols reduced OHSS incidence (RR: 0.25, 95% CI: 0.08, 0.79) and adverse event rates (RR: 0.36, 95% CI: 0.19, 0.67).

### Non-significant effects

Several integrated interventions showed no statistically significant effects for specific outcomes in women with PCOS complicated by infertility (Table 1). Compound Xuanju capsules combined with clomiphene showed no significant effects on pregnancy rate or cycle ovulation rate, while Compound Xuanju capsules combined with letrozole did not significantly improve pregnancy rate. Kidney-tonifying and yang-invigorating Chinese herbal therapies combined with Western ovulation-induction drugs showed no significant effects on follicle-stimulating hormone or estradiol levels. Chinese patent medicines or Chinese herbal decoctions combined with Western medicine did not significantly reduce spontaneous abortion rates. Kidney-tonifying and menstruation-regulating formulas combined with ovulation-inducing drugs showed no significant improvement in estradiol or follicle-stimulating hormone levels. Jinfeng Wan combined with Western medicine did not significantly improve luteinizing hormone levels or ovulation rates. Kuntai capsules combined with clomiphene showed no significant improvement in ovulation rate, and Kuntai capsules combined with clomiphene plus HCG showed no significant effects on ovulation rate or pregnancy rate. Kuntai capsules combined with ethinylestradiol–cyproterone acetate did not significantly improve pregnancy rate. In assisted reproductive technology settings, IVF-ET combined with Chinese herbal medicine showed no significant effect on abortion rate, and IVF-ET combined with Chinese herbal medicine administered throughout the entire IVF process or during frozen embryo transfer did not significantly improve clinical pregnancy rate. In addition, acupuncture combined with Western medicine showed no significant effect on follicle-stimulating hormone levels.

### Heterogeneity

Reanalysis showed that significant heterogeneity was present in 33% of the assessed outcomes, indicated by I^2^values exceeding 50% or Cochran’s Q-test *p*-values below 0.1. This heterogeneity may be related to differences in age distribution, infertility duration, disease severity, herbal prescriptions, intervention strategies, sample sizes, outcome assessment methods, statistical approaches, treatment duration, and traditional Chinese medicine syndrome differentiation. Both clinical and methodological heterogeneity were involved. Heterogeneity assessment results were not reported in 6% of the included studies (Table 1).

### Assessment of risk of bias

Publication bias was detected in 48.4% of the outcomes. It was linked to several aspects such as small sample sizes, poor methodology, and lack of information on randomization, blinding, and allocation concealment. Other sources of study bias were incomplete reporting of withdrawals, follow-up, and intention-to-treat analysis, inadequate inclusion of gray literature and English-language literature, missing negative or null results, incomplete estimation of the sample size, and inconsistent efficacy measures across studies. Regarding the remaining outcomes, no significant publication bias was found, or bias assessment was not reported (51.6% of outcomes) (Table 1).

### AMSTAR score

The overall scores of the identified outcomes in terms of AMSTAR had a median of 6 and a range of 4 to 9. Supplementary Table S4 displays AMSTAR scores related to individual outcomes.

### GRADE rating and evidence classification

There was a high-grade rating in 29.4%, a moderate GRADE rating in 60.1%, and a low-grade rating in 10.5% of the evaluated samples. This dispersion was linked to small sample sizes, the occurrence of publication bias, and heterogeneity as suggested by I^2^ values of more than 50% (Supplementary Table S5). For evidence classification, four outcomes from two intervention types met Class I–III criteria. Chinese medicine plus Western medicine using the TCM Cycle Method showed Class I evidence for pregnancy rate (OR: 2.76, 95% CI: 2.43, 3.13) and Class II evidence for total clinical efficiency (OR: 3.78, 95% CI: 3.29, 4.34) and ovulation rate (OR: 2.90, 95% CI: 2.39, 3.50). TCM cycle therapy plus ovulation induction showed Class III evidence for ovulation rate (RR: 1.10, 95% CI: 1.06, 1.14). The remaining 131 statistically significant outcomes were classified as Class IV evidence, while 18 outcomes were categorized as non-significant (Table 2).

**Table 2:** Criteria for evidence classification.

Study	Disease	Intervention	Outcome	Evidence
Zhou Kunyan, 2014 (48)	Infertility with polycystic ovary syndrome	Compound Xuanju capsules + clomiphene citrate	The pregnancy rate	Class NS
Zhou Kunyan, 2014 (48)	Infertility with polycystic ovary syndrome	Compound Xuanju capsules + clomiphene citrate	Ovulation rate during the cycle	Class NS
Zhou Kunyan, 2014 (48)	Infertility with polycystic ovary syndrome	Compound Xuanju capsules + clomiphene citrate	Menstrual improvement rate	Class IV
Zhou Kunyan, 2014 (48)	Infertility with polycystic ovary syndrome	Compound Xuanju capsules + clomiphene citrate	T	Class IV
Zhou Kunyan, 2014 (48)	Infertility with polycystic ovary syndrome	Compound Xuanju capsules + letrozole	The pregnancy rate	Class NS
Zhou Kunyan, 2014 (48)	Infertility with polycystic ovary syndrome	Compound Xuanju capsules + letrozole	Ovulation rate during the cycle	Class IV
Zhou Kunyan, 2014 (48)	Infertility with polycystic ovary syndrome	Compound Xuanju capsules + letrozole	Menstrual improvement rate	Class IV
Zhou Kunyan, 2014 (48)	Infertility with polycystic ovary syndrome	Compound Xuanju capsules + letrozole	BMI	Class IV
Qianwen Ma, 2023 (31)	Polycystic ovary syndrome-related infertility	Compound Xuanju capsules + hormone	The ovulation rate	Class IV
Qianwen Ma, 2023 (31)	Polycystic ovary syndrome-related infertility	Compound Xuanju capsules + hormone	The pregnancy rate	Class IV
Qianwen Ma, 2023 (31)	Polycystic ovary syndrome-related infertility	Compound Xuanju capsules + hormone	LH level	Class IV
Qianwen Ma, 2023 (31)	Polycystic ovary syndrome-related infertility	Compound Xuanju capsules + hormone	FSH level	Class IV
Qianwen Ma, 2023 (31)	Polycystic ovary syndrome-related infertility	Compound Xuanju capsules + hormone	E2 level	Class IV
Qianwen Ma, 2023 (31)	Polycystic ovary syndrome-related infertility	Compound Xuanju capsules + hormone	LH/FSH ratio	Class IV
Qianwen Ma, 2023 (31)	Polycystic ovary syndrome-related infertility	Compound Xuanju capsules + hormone	T level	Class IV
Qianwen Ma, 2023 (31)	Polycystic ovary syndrome-related infertility	Compound Xuanju capsules + hormone	Endometrial thickness	Class IV
Qianwen Ma, 2023 (31)	Polycystic ovary syndrome-related infertility	Compound Xuanju capsules + hormone	Overall rate of effective treatment	Class IV
Qianwen Ma, 2023 (31)	Polycystic ovary syndrome-related infertility	Compound Xuanju capsules + hormone	Overall rate of effective Chinese Medicine-based treatment	Class IV
Qianwen Ma, 2023 (31)	Polycystic ovary syndrome-related infertility	Compound Xuanju capsules + hormone	BBT	Class IV
Qianwen Ma, 2023 (31)	Polycystic ovary syndrome-related infertility	Compound Xuanju capsules + hormone	Left antral follicle count	Class IV
Qianwen Ma, 2023 (31)	Polycystic ovary syndrome-related infertility	Compound Xuanju capsules + hormone	Right antral follicle count	Class IV
Qianwen Ma, 2023 (31)	Polycystic ovary syndrome-related infertility	Compound Xuanju capsules + hormone	Left ovarian volume	Class IV
Qianwen Ma, 2023 (31)	Polycystic ovary syndrome-related infertility	Compound Xuanju capsules + hormone	Right ovarian volume	Class IV
Qianwen Ma, 2023 (31)	Polycystic ovary syndrome-related infertility	Compound Xuanju capsules + hormone	Follicle count	Class IV
Qianwen Ma, 2023 (31)	Polycystic ovary syndrome-related infertility	Compound Xuanju capsules + hormone	Maximum follicle diameter	Class IV
Qianwen Ma, 2023 (31)	Polycystic ovary syndrome-related infertility	Compound Xuanju capsules + hormone	HGF	Class IV
Qianwen Ma, 2023 (31)	Polycystic ovary syndrome-related infertility	Compound Xuanju capsules + hormone	VEGF	Class IV
Du Xiu, 2020 (36)	Infertility with PCOS	Compound Xuanju capsules + Western medicine	The pregnancy rate	Class IV
Du Xiu, 2020 (36)	Infertility with PCOS	Compound Xuanju capsules + Western medicine	Ovulation rate during the cycle	Class IV
Du Xiu, 2020 (36)	Infertility with PCOS	Compound Xuanju capsules + Western medicine	Comparison of the improvement of TCM symptoms	Class IV
Du Xiu, 2020 (36)	Infertility with PCOS	Compound Xuanju capsules + Western medicine	T	Class IV
Du Xiu, 2020 (36)	Infertility with PCOS	Compound Xuanju capsules + Western medicine	Endometrial thickness	Class IV
Zhao Shuai, 2022 (47)	Infertility caused by polycystic ovary syndrome	Traditional Chinese medicine for tonifying the kidney and enhancing Yang + Western medicine for inducing ovulation	Total effective rate	Class IV
Zhao Shuai, 2022 (47)	Infertility caused by polycystic ovary syndrome	Traditional Chinese medicine for tonifying the kidney and enhancing Yang + Western medicine for inducing ovulation	The pregnancy rate	Class IV
Zhao Shuai, 2022 (47)	Infertility caused by polycystic ovary syndrome	Traditional Chinese medicine for tonifying the kidney and enhancing Yang + Western medicine for inducing ovulation	Luteinizing hormone	Class IV
Zhao Shuai, 2022 (47)	Infertility caused by polycystic ovary syndrome	Traditional Chinese medicine for tonifying the kidney and enhancing Yang + Western medicine for inducing ovulation	Follicle-stimulating hormone	Class NS
Zhao Shuai, 2022 (47)	Infertility caused by polycystic ovary syndrome	Traditional Chinese medicine for tonifying the kidney and enhancing Yang + Western medicine for inducing ovulation	Luteinizing hormone/follicle-stimulating hormone	Class IV
Zhao Shuai, 2022 (47)	Infertility caused by polycystic ovary syndrome	Traditional Chinese medicine for tonifying the kidney and enhancing Yang + Western medicine for inducing ovulation	Estradiol	Class NS
Zhao Shuai, 2022 (47)	Infertility caused by polycystic ovary syndrome	Traditional Chinese medicine for tonifying the kidney and enhancing Yang + Western medicine for inducing ovulation	T	Class IV
Zhao Shuai, 2022 (47)	Infertility caused by polycystic ovary syndrome	Traditional Chinese medicine for tonifying the kidney and enhancing Yang + Western medicine for inducing ovulation	Endometrial thickness	Class IV
Zhao Shuai, 2022 (47)	Infertility caused by polycystic ovary syndrome	Traditional Chinese medicine for tonifying the kidney and enhancing Yang + Western medicine for inducing ovulation	Endometrial spiral artery pulsatility index	Class IV
Zhao Shuai, 2022 (47)	Infertility caused by polycystic ovary syndrome	Traditional Chinese medicine for tonifying the kidney and enhancing Yang + Western medicine for inducing ovulation	Endometrial spiral artery resistance index	Class IV
Yang Ting, 2022 (46)	Infertility with polycystic ovary syndrome	Traditional Chinese medicine/(traditional Chinese medicine + acupuncture) + conventional Western medicine	Comprehensive clinical efficacy	Class IV
Yang Ting, 2022 (46)	Infertility with polycystic ovary syndrome	Traditional Chinese medicine/(traditional Chinese medicine + acupuncture) + conventional Western medicine	The pregnancy rate	Class IV
Yang Ting, 2022 (46)	Infertility with polycystic ovary syndrome	Traditional Chinese medicine/(traditional Chinese medicine + acupuncture) + conventional Western medicine	Bad rectification rate	Class IV
Yang Ting, 2022 (46)	Infertility with polycystic ovary syndrome	Traditional Chinese medicine/(traditional Chinese medicine + acupuncture) + conventional Western medicine	The abortion rate	Class IV
Yang Ting, 2022 (46)	Infertility with polycystic ovary syndrome	Traditional Chinese medicine/(traditional Chinese medicine + acupuncture) + conventional Western medicine	The ovulation rate	Class IV
Yang Ting, 2022 (46)	Infertility with polycystic ovary syndrome	Traditional Chinese medicine/(traditional Chinese medicine + acupuncture) + conventional Western medicine	LH level	Class IV
Yang Ting, 2022 (46)	Infertility with polycystic ovary syndrome	Traditional Chinese medicine/(traditional Chinese medicine + acupuncture) + conventional Western medicine	LH/FSH	Class IV
Yang Ting, 2022 (46)	Infertility with polycystic ovary syndrome	Traditional Chinese medicine/(traditional Chinese medicine + acupuncture) + conventional Western medicine	T level	Class IV
Yang Ting, 2022 (46)	Infertility with polycystic ovary syndrome	Traditional Chinese medicine/(traditional Chinese medicine + acupuncture) + conventional Western medicine	Endometrial thickness	Class IV
Yang Ting, 2022 (46)	Infertility with polycystic ovary syndrome	Traditional Chinese medicine/(traditional Chinese medicine + acupuncture) + conventional Western medicine	Endometrial spiral artery pulsatility index	Class IV
Yang Ting, 2022 (46)	Infertility with polycystic ovary syndrome	Traditional Chinese medicine/(traditional Chinese medicine + acupuncture) + conventional Western medicine	Endometrial spiral artery resistance index	Class IV
Yang Ting, 2022 (46)	Infertility with polycystic ovary syndrome	Traditional Chinese medicine/(traditional Chinese medicine + acupuncture) + conventional Western medicine	TNF-α	Class IV
Huang Shouqia, 2021 (37)	PCOS infertility	Acupuncture + Western medicine	The pregnancy rate	Class IV
Huang Shouqia, 2021 (37)	PCOS infertility	Acupuncture + Western medicine	Ovulation rate during the cycle	Class IV
Huang Shouqia, 2021 (37)	PCOS infertility	Acupuncture + Western medicine	The ovulation rate	Class IV
Huang Shouqia, 2021 (37)	PCOS infertility	Acupuncture + Western medicine	FSH	Class IV
Huang Shouqia, 2021 (37)	PCOS infertility	Acupuncture + Western medicine	LH	Class IV
Huang Shouqia, 2021 (37)	PCOS infertility	Acupuncture + Western medicine	LH/FSH	Class IV
Huang Shouqia, 2021 (37)	PCOS infertility	Acupuncture + Western medicine	T	Class IV
Xue Zhu, 2022 (45)	PCOS infertility	Chinese patent medicines/Chinese herbal decoctions + Western medicines	Total clinical effective rate	Class IV
Xue Zhu, 2022 (45)	PCOS infertility	Chinese patent medicine + Western medicine	Total clinical effective rate	Class IV
Xue Zhu, 2022 (45)	PCOS infertility	Chinese herbal decoctions + Western medicine	Total clinical effective rate	Class IV
Xue Zhu, 2022 (45)	PCOS infertility	Chinese patent medicines/Chinese herbal decoctions + Western medicines	The ovulation rate	Class IV
Xue Zhu, 2022 (45)	PCOS infertility	Chinese patent medicines/Chinese herbal decoctions + Western medicines	The pregnancy rate	Class IV
Xue Zhu, 2022 (45)	PCOS infertility	Chinese patent medicine + Western medicine	The pregnancy rate	Class IV
Xue Zhu, 2022 (45)	PCOS infertility	Chinese herbal decoctions + Western medicine	The pregnancy rate	Class IV
Xue Zhu, 2022 (45)	PCOS infertility	Chinese patent medicines/Chinese herbal decoctions + Western medicines	LH	Class IV
Xue Zhu, 2022 (45)	PCOS infertility	Chinese patent medicines/Chinese herbal decoctions + Western medicines	FSH	Class IV
Xue Zhu, 2022 (45)	PCOS infertility	Chinese patent medicines/Chinese herbal decoctions + Western medicines	LH/FSH	Class IV
Xue Zhu, 2022 (45)	PCOS infertility	Chinese patent medicines/Chinese herbal decoctions + Western medicines	T	Class IV
Xue Zhu, 2022 (45)	PCOS infertility	Chinese patent medicines/Chinese herbal decoctions + Western medicines	Endometrial thickness	Class IV
Xue Zhu, 2022 (45)	PCOS infertility	Chinese patent medicines/Chinese herbal decoctions + Western medicines	The rate of spontaneous abortion	Class NS
Xu Lifang, 2018 (44)	Infertility caused by polycystic ovary syndrome	Reinforcing kidney and activating blood Chinese herbal + Western medicine	Total effective rate	Class IV
Xu Lifang, 2018 (44)	Infertility caused by polycystic ovary syndrome	Reinforcing kidney and activating blood Chinese herbal + Western medicine	The pregnancy rate	Class IV
Xu Lifang, 2018 (44)	Infertility caused by polycystic ovary syndrome	Reinforcing kidney and activating blood Chinese herbal + Western medicine	Security analysis	Class IV
Sheng Nan, 2021 (41)	Infertility in polycystic ovary	Reinforcing kidney and activating blood Chinese herbal + letrozole	Total effective rate	Class IV
Sheng Nan, 2021 (41)	Infertility in polycystic ovary	Reinforcing kidney and activating blood Chinese herbal + letrozole	The pregnancy rate	Class IV
Sheng Nan, 2021 (41)	Infertility in polycystic ovary	Reinforcing kidney and activating blood Chinese herbal + letrozole	Endometrial thickness	Class IV
Sheng Nan, 2021 (41)	Infertility in polycystic ovary	Reinforcing kidney and activating blood Chinese herbal + letrozole	LH	Class IV
Sheng Nan, 2021 (41)	Infertility in polycystic ovary	Reinforcing kidney and activating blood Chinese herbal + letrozole	FSH	Class IV
Chen Jing, 2021 (34)	Polycystic ovary syndrome infertility	Bushen Tiaojing recipes + ovulation induction drugs	The ovulation rate	Class IV
Chen Jing, 2021 (34)	Polycystic ovary syndrome infertility	Bushen Tiaojing recipes + ovulation induction drugs	The pregnancy rate	Class IV
Chen Jing, 2021 (34)	Polycystic ovary syndrome infertility	Bushen Tiaojing recipes + ovulation induction drugs	Incidence of adverse reactions	Class IV
Chen Jing, 2021 (34)	Polycystic ovary syndrome infertility	Bushen Tiaojing recipes + ovulation induction drugs	T	Class IV
Chen Jing, 2021 (34)	Polycystic ovary syndrome infertility	Bushen Tiaojing recipes + ovulation induction drugs	LH	Class IV
Chen Jing, 2021 (34)	Polycystic ovary syndrome infertility	Bushen Tiaojing recipes + ovulation induction drugs	FSH	Class NS
Chen Jing, 2021 (34)	Polycystic ovary syndrome infertility	Bushen Tiaojing recipes + ovulation induction drugs	Estradiol	Class NS
Chen Jing, 2021 (34)	Polycystic ovary syndrome infertility	Bushen Tiaojing recipes + ovulation induction drugs	Endometrial thickness	Class IV
Chen Jing, 2021 (34)	Polycystic ovary syndrome infertility	Bushen Tiaojing recipes + ovulation induction drugs	Ovarian volume	Class IV
DING Ke-fan, 2024 (35)	Infertility with PCOS	Dingkundan + letrozole	Endometrial thickness	Class IV
DING Ke-fan, 2024 (35)	Infertility with PCOS	Dingkundan + letrozole	The ovulation rate	Class IV
DING Ke-fan, 2024 (35)	Infertility with PCOS	Dingkundan + letrozole	The pregnancy rate	Class IV
DING Ke-fan, 2024 (35)	Infertility with PCOS	Dingkundan + other Western medicines	Endometrial thickness	Class IV
DING Ke-fan, 2024 (35)	Infertility with PCOS	Dingkundan + other Western medicines	The ovulation rate	Class IV
DING Ke-fan, 2024 (35)	Infertility with PCOS	Dingkundan + other Western medicines	The pregnancy rate	Class IV
DING Ke-fan, 2024 (35)	Infertility with PCOS	Dingkundan + Western medicine	Endometrial thickness	Class IV
DING Ke-fan, 2024 (35)	Infertility with PCOS	Dingkundan + Western medicine	The number of mature follicles	Class IV
DING Ke-fan, 2024 (35)	Infertility with PCOS	Dingkundan + Western medicine	The ovulation rate	Class IV
DING Ke-fan, 2024 (35)	Infertility with PCOS	Dingkundan + Western medicine	The pregnancy rate	Class IV
DING Ke-fan, 2024 (35)	Infertility with PCOS	Dingkundan + Western medicine	LH	Class IV
DING Ke-fan, 2024 (35)	Infertility with PCOS	Dingkundan + Western medicine	Total effective rate	Class IV
Yu Xu, 2022 (32)	Infertility in patients with polycystic ovary syndrome	Jinfeng pills + Western medicine	The effective rate	Class IV
Yu Xu, 2022 (32)	Infertility in patients with polycystic ovary syndrome	Jinfeng pills + Western medicine	FSH	Class IV
Yu Xu, 2022 (32)	Infertility in patients with polycystic ovary syndrome	Jinfeng pills + Western medicine	LH	Class NS
Yu Xu, 2022 (32)	Infertility in patients with polycystic ovary syndrome	Jinfeng pills + Western medicine	E2	Class IV
Yu Xu, 2022 (32)	Infertility in patients with polycystic ovary syndrome	Jinfeng pills + Western medicine	T	Class IV
Yu Xu, 2022 (32)	Infertility in patients with polycystic ovary syndrome	Jinfeng pills + Western medicine	The ovulation rate	Class NS
Yu Xu, 2022 (32)	Infertility in patients with polycystic ovary syndrome	Jinfeng pills + Western medicine	The pregnancy rate	Class IV
Liu Ying, 2019 (38)	Polycystic ovary syndrome infertility	Kuntai capsules + clomiphene citrate	The pregnancy rate	Class IV
Liu Ying, 2019 (38)	Polycystic ovary syndrome infertility	Kuntai capsules + clomiphene citrate	The ovulation rate	Class NS
Liu Ying, 2019 (38)	Polycystic ovary syndrome infertility	Kuntai capsules + clomiphene citrate + HCG	The pregnancy rate	Class NS
Liu Ying, 2019 (38)	Polycystic ovary syndrome infertility	Kuntai capsules + clomiphene citrate + HCG	The ovulation rate	Class NS
Liu Ying, 2019 (38)	Polycystic ovary syndrome infertility	Kuntai capsules + ethinylestradiol and cyproterone acetate tablets + clomiphene citrate + HMG	The pregnancy rate	Class IV
Liu Ying, 2019 (38)	Polycystic ovary syndrome infertility	Kuntai capsules + ethinylestradiol and cyproterone acetate tablets + clomiphene citrate + HMG	The ovulation rate	Class IV
Liu Ying, 2019 (38)	Polycystic ovary syndrome infertility	Kuntai capsules + letrozole	The pregnancy rate	Class IV
Liu Ying, 2019 (38)	Polycystic ovary syndrome infertility	Kuntai capsules + letrozole	The ovulation rate	Class IV
Liu Ying, 2019 (38)	Polycystic ovary syndrome infertility	Kuntai capsules + ethinylestradiol and cyproterone acetate tablets	The pregnancy rate	Class NS
Liu Ying, 2019 (38)	Polycystic ovary syndrome infertility	Kuntai capsules + ethinylestradiol and cyproterone acetate tablets	The ovulation rate	Class IV
Liu Ying, 2019 (38)	Polycystic ovary syndrome infertility	Kuntai capsules + Western medicine	The pregnancy rate	Class IV
Liu Ying, 2019 (38)	Polycystic ovary syndrome infertility	Kuntai capsules + Western medicine	The ovulation rate	Class IV
Chang Liu, 2021 (30)	Infertility with PCOS	A combination of IVF-ET and CHM	The clinical pregnancy rate	Class IV
Chang Liu, 2021 (30)	Infertility with PCOS	A combination of IVF-ET and CHM	The abortion rate	Class NS
Chang Liu, 2021 (30)	Infertility with PCOS	A combination of IVF-ET and CHM	The OHSS incidence.	Class IV
Chang Liu, 2021 (30)	Infertility with PCOS	A combination of IVF-ET and CHM (Decoction)	The clinical pregnancy rate	Class IV
Chang Liu, 2021 (30)	Infertility with PCOS	A combination of IVF-ET and CHM (Granules)	The clinical pregnancy rate	Class IV
Chang Liu, 2021 (30)	Infertility with PCOS	A combination of IVF-ET and CHM (Decoction + Granules)	The clinical pregnancy rate	Class IV
Chang Liu, 2021 (30)	Infertility with PCOS	A combination of IVF-ET and CHM (before oocyte extraction)	The clinical pregnancy rate	Class IV
Chang Liu, 2021 (30)	Infertility with PCOS	A combination of IVF-ET and CHM (over all the process of IVF)	The clinical pregnancy rate	Class NS
Chang Liu, 2021 (30)	Infertility with PCOS	A combination of IVF-ET (frozen embryo) and CHM	The clinical pregnancy rate	Class NS
Chang Liu, 2021 (30)	Infertility with PCOS	A combination of IVF-ET (fresh embryo) and CHM	The clinical pregnancy rate	Class IV
Chang Liu, 2021 (30)	Infertility with PCOS	A combination of IVF-ET (frozen and fresh embryo) and CHM	The clinical pregnancy rate	Class IV
Chang Liu, 2021 (30)	Infertility with PCOS	A combination of IVF-ET (fresh embryo, 2–3 days transfer) and CHM	The clinical pregnancy rate	Class IV
Chang Liu, 2021 (30)	Infertility with PCOS	A combination of IVF-ET (fresh embryo, 18–22 h transfer) and CHM	The clinical pregnancy rate	Class IV
Chang Liu, 2021 (30)	Infertility with PCOS	A combination of IVF-ET (fresh embryo, 2–3 days transfer and fresh embryo, 18–22 h transfer) and CHM	The clinical pregnancy rate	Class IV
Cai Shu, 2016 (29)	PCOS infertility	Combine traditional Chinese and Western medicine	The ovulation rate	Class IV
Cai Shu, 2016 (29)	PCOS infertility	Combine traditional Chinese and Western medicine	The pregnancy rate	Class IV
Cai Shu, 2016 (29)	PCOS infertility	Combine traditional Chinese and Western medicine	Effective rate	Class IV
Wu Haiyan, 2019 (42)	Infertility with PCOS	Chinese medicine + Western medicine (Traditional Chinese Medicine Cycle Method)	Total clinical efficiency	Class II
Wu Haiyan, 2019 (42)	Infertility with PCOS	Chinese medicine + Western medicine (Traditional Chinese Medicine Cycle Method)	The ovulation rate	Class II
Wu Haiyan, 2019 (42)	Infertility with PCOS	Chinese medicine + Western medicine (Traditional Chinese Medicine Cycle Method)	The pregnancy rate	Class I
Wu Haiyan, 2019 (42)	Infertility with PCOS	Acupuncture + Western medicine (Traditional Chinese Medicine Cycle Method)	Total clinical efficiency	Class IV
Wu Haiyan, 2019 (42)	Infertility with PCOS	Acupuncture + Western medicine (Traditional Chinese Medicine Cycle Method)	The ovulation rate	Class IV
Wu Haiyan, 2019 (42)	Infertility with PCOS	Acupuncture + Western medicine (Traditional Chinese Medicine Cycle Method)	The pregnancy rate	Class IV
Li Huimin, 2024 (39)	Infertility caused by polycystic ovary syndrome	Traditional Chinese medicine cycle therapy + ovulation induction protocol	The pregnancy rate	Class IV
Li Huimin, 2024 (39)	Infertility caused by polycystic ovary syndrome	Traditional Chinese medicine cycle therapy + ovulation induction protocol	Total effective rate	Class IV
Li Huimin, 2024 (39)	Infertility caused by polycystic ovary syndrome	Traditional Chinese medicine cycle therapy + ovulation induction protocol	The ovulation rate	Class III
Li Huimin, 2024 (39)	Infertility caused by polycystic ovary syndrome	Traditional Chinese medicine cycle therapy + ovulation induction protocol	Androgen level	Class IV
Li Huimin, 2024 (39)	Infertility caused by polycystic ovary syndrome	Traditional Chinese medicine cycle therapy + ovulation induction protocol	LH	Class IV
Li Huimin, 2024 (39)	Infertility caused by polycystic ovary syndrome	Traditional Chinese medicine cycle therapy + ovulation induction protocol	Endometrial thickness	Class IV
Li Huimin, 2024 (39)	Infertility caused by polycystic ovary syndrome	Traditional Chinese medicine cycle therapy + ovulation induction protocol	Incidence of OHSS	Class IV
Li Huimin, 2024 (39)	Infertility caused by polycystic ovary syndrome	Traditional Chinese medicine cycle therapy + ovulation induction protocol	Adverse reaction rate	Class IV

CC: clomiphene citrate; LE: letrozole; HCG: human chorionic gonadotropin; HMG: urogonadotropin; CHM: Chinese herbal medicine; IVF-ET: in vitro fertilization-embryo transfer; T: testosterone; BMI: body mass index; LH: luteinizing hormone; FSH: follicle-stimulating hormone; E2: estradiol; BBT: basal body temperature; HGF: hepatocyte growth factor; VEGF: vascular endothelial growth factor; TNF-α: tumor necrosis factor-α; OHSS: ovarian hyper-stimulation syndrome.

## Discussion

In this umbrella meta-analysis, we summarized evidence from existing meta-analyses of integrated traditional Chinese and Western medicine for PCOS-related infertility. Although many outcomes favored integrated treatment with statistically significant results, their interpretation should depend primarily on evidence quality and credibility rather than statistical significance alone. Among the 153 outcomes assessed, only four met the criteria for Class I–III evidence, whereas most were classified as Class IV or showed no significant effect, indicating that the positive findings did not constitute strong or conclusive evidence. The reliability of the findings was weakened by small sample sizes, clinical and methodological heterogeneity, possible publication bias, and incomplete methodological reporting in both the original randomized trials and the included meta-analyses. Overall, integrated therapy may have potential benefits, but the current evidence does not provide definitive evidence of superiority over Western medicine alone.

Non-significant findings further supported a cautious interpretation. Although many outcomes favored integrated therapy, 18 outcomes showed no statistically significant effect, involving clinically relevant outcomes such as pregnancy rate, ovulation rate, endocrine indicators, miscarriage- or abortion-related outcomes, and clinical pregnancy rate in some assisted reproductive technology settings. These null findings suggest that treatment effects may vary across intervention types and outcome domains and that positive results should not be generalized across all integrative interventions. Taken together, the evidence suggests promising associations with selected outcomes, but robust evidence remains limited.

The noted advantages of integrative interventions are related to the present-day perspectives on infertility in PCOS as a multisystem complication. It has been indicated that reproductive dysfunction in women with PCOS is not only associated with impaired ovulation but also with metabolic dysfunction, endocrine disruption related to obesity, long-term low-grade inflammation, oxidative stress, and immune dysregulation (49–51). Overweight status and insulin resistance worsen hyperandrogenism and follicular dysfunction and reduce endometrial receptivity and implantation capacity (51). Traditional ovulation stimulation primarily acts on hypothalamic–pituitary–ovarian axis disruptions and may not adequately cover these whole-body and uterine processes. In comparison, interventions based on traditional Chinese medicine have been reported to have multi-target regulatory effects such as regulation of sex steroid receptor expression, improved insulin sensitivity, weakened inflammatory signaling, and strengthening uterine microcirculation (52–54). This umbrella review found significant improvements in endometrial-related parameters with different integrative modalities, indicating the potential for improving uterine receptivity along with ovulation restoration.

Endometrial dysfunction is beginning to get attention as a factor in infertility and early pregnancy loss in women with PCOS. The results of experimental and clinical trials indicate that progesterone resistance, disturbances in immune cells, and the continuing inflammatory stimulation of the endometrial environment prevent implantation and development of the placenta (55, 56). More recent data indicate that dysregulated endometrial immune responses, especially involving T-cell subsets and cytokine signaling, may predispose women with PCOS to implantation failure and miscarriage (57). This umbrella meta-analysis combines endocrine, endometrial, and pregnancy outcomes, unlike earlier reviews that examined their effects in isolation and in separate analytical frameworks, which enables greater interpretation of the effects. Evidence on the gut–endocrine–reproductive axis also indicates that dysbiosis of gut microbiota may contribute to the augmentation of metabolic and inflammatory imbalances in PCOS, and thereby indirectly influence endometrial function and fertility rates (58), which supports the biological plausibility of multi-target integrative approaches.

The other domain of significance that is addressed by this study is safety. Women with PCOS receiving fertility therapy, especially ART, are at risk of OHSS and other treatment-associated complications (59). Although in a number of previous syntheses, efficacy was predominantly the primary focus, the current umbrella meta-analysis incorporates safety endpoints in a variety of integrative interventions. The results indicate that certain mixed strategies are associated with fewer cases of OHSS and adverse events. Acupuncture and other adjunctive therapies can have an effect on the regulation of the autonomic nervous system, oxidative stress, and ovarian blood flow and thus may reduce excessive ovarian responses during stimulation cycles (60). Such results support the use of integrative approaches to promote safer ovarian stimulation and more personalized ART regimens in women with PCOS (61).

One of the methodological strengths of this umbrella review is the clear evaluation of the credibility of evidence. Several meta-analyses conducted in the past also provided pooled estimates of effects without an in-depth analysis of heterogeneity, publication bias, or methodology. This study diminishes the chances of overinterpretation and enhances transparency through the adoption of an umbrella review framework that differentiates between statistically significant results and those with more substantial evidence. This is in line with the current suggestions on overviews of systematic reviews, as well as with the prudent application of integrative medicine research to clinical practice (18). New progress in the precise stratification of PCOS phenotypes also highlights the necessity of identifying patient subgroups that are most likely to benefit from a given integrative intervention, which needs to be investigated further (62).

Several limitations should be considered when interpreting these findings. Although the included meta-analyses were based on randomized controlled trials, many primary studies were small and inadequately reported key methodological details, including randomization, allocation concealment, blinding, attrition, and follow-up, which lowered evidence certainty. Clinical and methodological heterogeneity was evident across herbal prescriptions, acupuncture protocols, cycle-based strategies, treatment duration, Western medicine comparators, and outcome definitions, limiting identification of which integrative interventions may benefit particular patient subgroups. In addition, grouping diverse interventions under the broad category of integrated therapy may have obscured differences in effectiveness and safety across specific approaches, and further subgroup analyses were limited because this review was based on published meta-analyses. Publication bias in some outcomes suggested possible overrepresentation of favorable findings, while overlap among primary studies may have introduced duplicate evidence.

Live birth outcomes were also inadequately reported. Most included meta-analyses assessed surrogate or intermediate fertility indicators, including ovulation, clinical pregnancy, endocrine parameters, and endometrial thickness. Although clinically relevant, these measures cannot be assumed to correspond to improved live birth, the most important endpoint in infertility management. Therefore, improvements in ovulation, pregnancy rate, hormonal status, or endometrial receptivity should be interpreted cautiously when live birth evidence is limited. Future randomized trials and meta-analyses should include live birth, cumulative live birth, miscarriage, offspring health, and long-term maternal outcomes as key endpoints.

The generalizability of these findings beyond China may also be limited. Most evidence came from Chinese clinical settings, where TCM diagnostic approaches, practitioner training, herbal product standards, patient expectations, regulatory systems, and integration with Western reproductive medicine may differ from those in other countries. Therefore, these results require further validation before being applied to non-Chinese populations or healthcare systems. In addition, offspring health and long-term metabolic outcomes were insufficiently reported. Future multicenter randomized trials in diverse populations are needed to confirm the effectiveness, safety, and external validity of integrated treatment strategies for PCOS-related infertility.

## Conclusion

This umbrella meta-analysis suggests that integrated traditional Chinese and Western medicine shows promising associations with improvements in selected reproductive, endocrine, endometrial, and safety-related outcomes among women with PCOS-related infertility. However, robust evidence supporting these associations remains limited, and the findings should therefore be interpreted cautiously. Among 153 evaluated outcomes, only four met the criteria for Class I–III evidence, whereas most outcomes were classified as Class IV or showed no statistically significant effect. Therefore, the current evidence does not establish that integrated therapy is superior to Western medicine alone. The certainty of evidence was weakened by small study samples, heterogeneity in interventions and outcome measures, possible publication bias, and incomplete methodological reporting. Further well-designed multicenter randomized controlled trials with standardized integrative protocols, sufficient follow-up, and clinically meaningful endpoints, particularly live birth, are needed to confirm the effectiveness, safety, and generalizability of integrated treatment strategies for PCOS-related infertility.

## Data Availability

The original contributions presented in the study are included in the article/Supplementary material, further inquiries can be directed to the corresponding author/s.
