# Supplementary material for: The efficacy and safety of combined traditional Chinese and Western medicine treatment for PCOS-related infertility: an umbrella meta-analysis
Source: Front Med (Lausanne). 2026 Aug 6;13:1877844. doi: 10.3389/fmed.2026.1877844 (PMC13490117; doi:10.3389/fmed.2026.1877844)
Supplement: Supplementary file 1 [file Table_1.docx]

| **Table S1 The search strategy** | | |
| --- | --- | --- |
| **Datebases** | **Searching strategy** | **Literature number** |
| Pubmed | (((((((((((((((((Polycystic Ovary Syndrome[MeSH Terms]) OR (Polycystic Ovary Syndrome[Title/Abstract])) OR (Ovary Syndrome, Polycystic[Title/Abstract])) OR (Syndrome, Polycystic Ovary[Title/Abstract])) OR (Polycystic Ovarian Syndrome[Title/Abstract])) OR (Ovarian Syndrome, Polycystic[Title/Abstract])) OR (Polycystic Ovary Syndrome 1[Title/Abstract])) OR (Sclerocystic Ovarian Degeneration[Title/Abstract])) OR (Ovarian Degeneration, Sclerocystic[Title/Abstract])) OR (Sclerocystic Ovary Syndrome[Title/Abstract])) OR (Stein-Leventhal Syndrome[Title/Abstract])) OR (Stein Leventhal Syndrome[Title/Abstract])) OR (Syndrome, Stein-Leventhal[Title/Abstract])) OR (Sclerocystic Ovaries[Title/Abstract])) OR (Ovary, Sclerocystic[Title/Abstract])) OR (Sclerocystic Ovary[Title/Abstract])) AND ((((((((Infertility[MeSH Terms]) ) OR (Infertility[Title/Abstract])) OR (Sterility, Reproductive[Title/Abstract])) OR (Reproductive Sterility[Title/Abstract])) OR (Sterility[Title/Abstract])) OR (Subfertility[Title/Abstract])) OR (Sub-Fertility[Title/Abstract]))) AND ((meta-analysis[Title/Abstract]) OR (Systematic review[Title/Abstract])) | **264** |
| Web of science | 1 "TS=(Polycystic Ovary Syndrome OR Ovary Syndrome, Polycystic OR Syndrome, Polycystic Ovary OR Polycystic Ovarian Syndrome OR Ovarian Syndrome, Polycystic OR Polycystic Ovary Syndrome 1 OR Sclerocystic Ovarian Degeneration OR Ovarian Degeneration, Sclerocystic OR Sclerocystic Ovary Syndrome OR Stein-Leventhal Syndrome OR Stein Leventhal Syndrome OR Syndrome, Stein-Leventhal OR Sclerocystic Ovaries OR Ovary, Sclerocystic OR Sclerocystic Ovary) " 30713  2 "TS=(Infertility OR Sterility, Reproductive OR Reproductive Sterility OR Sterility OR Subfertility OR Sub-Fertility) " 117798  3 "TS=(meta-analysis OR Systematic review) " 759108  4 "#3 AND #2 AND #1 " 391 | **391** |
| Embase | #29 #17 AND #25 AND #28 505  #28 #26 OR #27 623092  #27 'systematic review':ab,ti 439511  #26 'meta-analysis':ab,ti 392789  #25 #18 OR #19 OR #20 OR #21 OR #22 OR #23 OR #24 218179  #24 'sub-fertility':ab,ti 585  #23 'sterility':ab,ti 22965  #22 'subfertility':ab,ti 7168  #21 'reproductive sterility':ab,ti 34  #20 'sterility, reproductive':ab,ti 2  #19 'infertility':ab,ti 109789  #18 'infertility'/exp 171521  #17 #1 OR #2 OR #3 OR #4 OR #5 OR #6 OR #7 OR #8 OR #9 OR #10 OR #11 OR #12 OR #13 OR #14 OR #15 OR #16 48895  #16 'sclerocystic ovary':ab,ti 44  #15 'ovary, sclerocystic':ab,ti 1  #14 'sclerocystic ovaries':ab,ti 46  #13 'syndrome, stein-leventhal':ab,ti 14  #12 'stein leventhal syndrome':ab,ti 651  #11 'stein-leventhal syndrome':ab,ti 652  #10 'sclerocystic ovary syndrome':ab,ti 35  #9 'ovarian degeneration, sclerocystic':ab,ti 0  #8 'sclerocystic ovarian degeneration':ab,ti 2  #7 'polycystic ovary syndrome 1':ab,ti 22  #6 'ovarian syndrome, polycystic':ab,ti 5  #5 'polycystic ovarian syndrome':ab,ti 7571  #4 'syndrome, polycystic ovary':ab,ti 66  #3 'ovary syndrome, polycystic':ab,ti 19  #2 'polycystic ovary syndrome':ab,ti 28397  #1 'polycystic ovary syndrome'/exp 45960 | **505** |
| Cochrane | #1 (Polycystic Ovary Syndrome):ti,ab,kw OR (Ovary Syndrome, Polycystic):ti,ab,kw OR (Syndrome, Polycystic Ovary):ti,ab,kw OR (Polycystic Ovarian Syndrome):ti,ab,kw OR (Ovarian Syndrome, Polycystic):ti,ab,kw 5389  #2 (Polycystic Ovary Syndrome 1):ti,ab,kw OR (Sclerocystic Ovarian Degeneration):ti,ab,kw OR (Ovarian Degeneration, Sclerocystic):ti,ab,kw OR (Sclerocystic Ovary Syndrome):ti,ab,kw OR (Stein-Leventhal Syndrome):ti,ab,kw 2644  #3 (Stein Leventhal Syndrome):ti,ab,kw OR (Syndrome, Stein-Leventhal):ti,ab,kw OR (Sclerocystic Ovaries):ti,ab,kw OR (Ovary, Sclerocystic):ti,ab,kw OR (Sclerocystic Ovary):ti,ab,kw 47  #4 #1 OR #2 OR #3 5392  #5 (Infertility):ti,ab,kw OR (Sterility, Reproductive):ti,ab,kw OR (Reproductive Sterility):ti,ab,kw OR (Sterility):ti,ab,kw OR (Subfertility):ti,ab,kw 12832  #6 (Sub-Fertility):ti,ab,kw 27  #7 #5 OR #6 12833  #8 (meta-analysis):ti,ab,kw OR (Systematic review):ti,ab,kw 30566  #9 #4 AND #7 AND #8 32 | **32** |
| CNKI | (TKA=多囊卵巢综合征 OR 硬化性囊性卵巢变性 OR 卵巢变性硬化性囊性 OR 硬化性囊性卵巢综合征 OR 斯坦-莱文索尔综合征 OR 综合征斯坦-莱文索尔 OR 硬化性囊性卵巢 OR 卵巢硬化性囊性) AND (TKA=不孕症 OR 不育症生殖 OR 生殖不育症 OR 不育 OR 亚不育 OR 亚不育症) | **9293** |
| Wanfang | 题名或关键词:(多囊卵巢综合征 OR 硬化性囊性卵巢变性 OR 卵巢变性硬化性囊性 OR 硬化性囊性卵巢综合征 OR 斯坦-莱文索尔综合征 OR 综合征斯坦-莱文索尔 OR 硬化性囊性卵巢 OR 卵巢硬化性囊性) AND 题名或关键词:(不孕症 OR 不育症生殖 OR 生殖不育症 OR 不育 OR 亚不育 OR 亚不育症 ) | **3340** |
| VIP | (K=(多囊卵巢综合征 OR 硬化性囊性卵巢变性 OR 卵巢变性硬化性囊性 OR 硬化性囊性卵巢综合征 OR 斯坦-莱文索尔综合征 OR 综合征斯坦-莱文索尔 OR 硬化性囊性卵巢 OR 卵巢硬化性囊性) OR R=(多囊卵巢综合征 OR 硬化性囊性卵巢变性 OR 卵巢变性硬化性囊性 OR 硬化性囊性卵巢综合征 OR 斯坦-莱文索尔综合征 OR 综合征斯坦-莱文索尔 OR 硬化性囊性卵巢 OR 卵巢硬化性囊性) OR T=(多囊卵巢综合征 OR 硬化性囊性卵巢变性 OR 卵巢变性硬化性囊性 OR 硬化性囊性卵巢综合征 OR 斯坦-莱文索尔综合征 OR 综合征斯坦-莱文索尔 OR 硬化性囊性卵巢 OR 卵巢硬化性囊性)) AND (K=(不孕症 OR 不育症生殖 OR 生殖不育症 OR 不育 OR 亚不育 OR 亚不育症) OR R=(不孕症 OR 不育症生殖 OR 生殖不育症 OR 不育 OR 亚不育 OR 亚不育症) OR T=(不孕症 OR 不育症生殖 OR 生殖不育症 OR 不育 OR 亚不育 OR 亚不育症)) | **3756** |
