# Supplementary material for: The efficacy and safety of combined traditional Chinese and Western medicine treatment for PCOS-related infertility: an umbrella meta-analysis
Source: Front Med (Lausanne). 2026 Aug 6;13:1877844. doi: 10.3389/fmed.2026.1877844 (PMC13490117; doi:10.3389/fmed.2026.1877844)
Supplement: Supplementary file 2 [file Table_2.docx]

| Table S2 Evidence categories criteria | |
| --- | --- |
| Evidence class | Description |
| Class I: convincing evidence | >1000 cases (or >20000 participants for continuous outcomes), statistical significance at P < 10−6 (random-effects), no evidence of small-study effects and excess significance bias; 95% prediction interval excluded the null, no large heterogeneity (I2 < 50%) |
| Class II: highly suggestive evidence | >1000 cases (or >20000 participants for continuous outcomes), statistical significance at P < 10−6 (random-effects) and largest study with 95% CI excluding the null value |
| Class III: suggestive evidence | >1000 cases (or >20000 participants for continuous outcomes) and statistical significance at P < 0.001 |
| Class IV: weak evidence | The remaining significant associations with P < 0.05 |
| NS: non-significant | P > 0.05 |
