# Supplementary material for: The efficacy and safety of combined traditional Chinese and Western medicine treatment for PCOS-related infertility: an umbrella meta-analysis
Source: Front Med (Lausanne). 2026 Aug 6;13:1877844. doi: 10.3389/fmed.2026.1877844 (PMC13490117; doi:10.3389/fmed.2026.1877844)
Supplement: Supplementary file 3 [file Table_3.docx]

**Table S3 Citation Matrix**

| **Research** | **Intervening measure** | | | | | | | | | | | | | | | | | | | | | | | | | | | | | | | | | | | |  |
| --- | --- | --- | --- | --- | --- | --- | --- | --- | --- | --- | --- | --- | --- | --- | --- | --- | --- | --- | --- | --- | --- | --- | --- | --- | --- | --- | --- | --- | --- | --- | --- | --- | --- | --- | --- | --- | --- |
|  | CXJC+CC | CXJC+LE | CXJC+Hormone | CXJC+WM | KYE-CHM+OI-WM | TCM±ACU+CWM | ACU+WM | CPM/CHD+WM | CPM+WM | CHD+WM | RKAB-CH+WM | RKAB-CH+LE | BTR+OID | DKD+LE | DKD+OWM | DKD+WM | JFP+WM | KTC+CC | KTC+EE/CPA+CC+HMG | KTC+LE | KTC+EE/CPA | KTC+WM | IVF-ET+CHM | IVF-ET+CHM(D) | IVF-ET+CHM(G) | IVF-ET+CHM(D+G) | IVF-ET+CHM(BOE) | IVF-ET(FE)+CHM | IVF-ET(FFE)+CHM | IVF-ET(FE,2-3DT)+CHM | IVF-ET(FE,18-22HT)+CHM | IVF-ET(FE,2-3DT+FE,18-22HT)+CHM | CTCWM | CM+WM(TCMCM) | ACU+WM(TCMCM) | TCMCT+OIP | |
| Zhou2014 | 1 | 1 | 0 | 0 | 0 | 0 | 0 | 0 | 0 | 0 | 0 | 0 | 0 | 0 | 0 | 0 | 0 | 0 | 0 | 0 | 0 | 0 | 0 | 0 | 0 | 0 | 0 | 0 | 0 | 0 | 0 | 0 | 0 | 0 | 0 | 0 | |
| Ma2023 | 0 | 0 | 1 | 0 | 0 | 0 | 0 | 0 | 0 | 0 | 0 | 0 | 0 | 0 | 0 | 0 | 0 | 0 | 0 | 0 | 0 | 0 | 0 | 0 | 0 | 0 | 0 | 0 | 0 | 0 | 0 | 0 | 0 | 0 | 0 | 0 | |
| Du2020 | 0 | 0 | 0 | 1 | 0 | 0 | 0 | 0 | 0 | 0 | 0 | 0 | 0 | 0 | 0 | 0 | 0 | 0 | 0 | 0 | 0 | 0 | 0 | 0 | 0 | 0 | 0 | 0 | 0 | 0 | 0 | 0 | 0 | 0 | 0 | 0 | |
| Zhao2022 | 0 | 0 | 0 | 0 | 1 | 0 | 0 | 0 | 0 | 0 | 0 | 0 | 0 | 0 | 0 | 0 | 0 | 0 | 0 | 0 | 0 | 0 | 0 | 0 | 0 | 0 | 0 | 0 | 0 | 0 | 0 | 0 | 0 | 0 | 0 | 0 | |
| Yang2022 | 0 | 0 | 0 | 0 | 0 | 1 | 0 | 0 | 0 | 0 | 0 | 0 | 0 | 0 | 0 | 0 | 0 | 0 | 0 | 0 | 0 | 0 | 0 | 0 | 0 | 0 | 0 | 0 | 0 | 0 | 0 | 0 | 0 | 0 | 0 | 0 | |
| Huang2021 | 0 | 0 | 0 | 0 | 0 | 0 | 1 | 0 | 0 | 0 | 0 | 0 | 0 | 0 | 0 | 0 | 0 | 0 | 0 | 0 | 0 | 0 | 0 | 0 | 0 | 0 | 0 | 0 | 0 | 0 | 0 | 0 | 0 | 0 | 0 | 0 | |
| Xue2022 | 0 | 0 | 0 | 0 | 0 | 0 | 0 | 1 | 1 | 1 | 0 | 0 | 0 | 0 | 0 | 0 | 0 | 0 | 0 | 0 | 0 | 0 | 0 | 0 | 0 | 0 | 0 | 0 | 0 | 0 | 0 | 0 | 0 | 0 | 0 | 0 | |
| Xu2018 | 0 | 0 | 0 | 0 | 0 | 0 | 0 | 0 | 0 | 0 | 1 | 0 | 0 | 0 | 0 | 0 | 0 | 0 | 0 | 0 | 0 | 0 | 0 | 0 | 0 | 0 | 0 | 0 | 0 | 0 | 0 | 0 | 0 | 0 | 0 | 0 | |
| Sheng2021 | 0 | 0 | 0 | 0 | 0 | 0 | 0 | 0 | 0 | 0 | 0 | 1 | 0 | 0 | 0 | 0 | 0 | 0 | 0 | 0 | 0 | 0 | 0 | 0 | 0 | 0 | 0 | 0 | 0 | 0 | 0 | 0 | 0 | 0 | 0 | 0 | |
| Chen2021 | 0 | 0 | 0 | 0 | 0 | 0 | 0 | 0 | 0 | 0 | 0 | 0 | 1 | 0 | 0 | 0 | 0 | 0 | 0 | 0 | 0 | 0 | 0 | 0 | 0 | 0 | 0 | 0 | 0 | 0 | 0 | 0 | 0 | 0 | 0 | 0 | |
| Ding2024 | 0 | 0 | 0 | 0 | 0 | 0 | 0 | 0 | 0 | 0 | 0 | 0 | 0 | 1 | 1 | 1 | 0 | 0 | 0 | 0 | 0 | 0 | 0 | 0 | 0 | 0 | 0 | 0 | 0 | 0 | 0 | 0 | 0 | 0 | 0 | 0 | |
| Yu2022 | 0 | 0 | 0 | 0 | 0 | 0 | 0 | 0 | 0 | 0 | 0 | 0 | 0 | 0 | 0 | 0 | 1 | 0 | 0 | 0 | 0 | 0 | 0 | 0 | 0 | 0 | 0 | 0 | 0 | 0 | 0 | 0 | 0 | 0 | 0 | 0 | |
| Liu2019 | 0 | 0 | 0 | 0 | 0 | 0 | 0 | 0 | 0 | 0 | 0 | 0 | 0 | 0 | 0 | 0 | 0 | 1 | 1 | 1 | 1 | 1 | 0 | 0 | 0 | 0 | 0 | 0 | 0 | 0 | 0 | 0 | 0 | 0 | 0 | 0 | |
| Liu2021 | 0 | 0 | 0 | 0 | 0 | 0 | 0 | 0 | 0 | 0 | 0 | 0 | 0 | 0 | 0 | 0 | 0 | 0 | 0 | 0 | 0 | 0 | 1 | 1 | 1 | 1 | 1 | 1 | 1 | 1 | 1 | 0 | 0 | 0 | 0 | 0 | |
| Cai2016 | 0 | 0 | 0 | 0 | 0 | 0 | 0 | 0 | 0 | 0 | 0 | 0 | 0 | 0 | 0 | 0 | 0 | 0 | 0 | 0 | 0 | 0 | 0 | 0 | 0 | 0 | 0 | 0 | 0 | 0 | 0 | 0 | 1 | 0 | 0 | 0 | |
| Wu2019 | 0 | 0 | 0 | 0 | 0 | 0 | 0 | 0 | 0 | 0 | 0 | 0 | 0 | 0 | 0 | 0 | 0 | 0 | 0 | 0 | 0 | 0 | 0 | 0 | 0 | 0 | 0 | 0 | 0 | 0 | 0 | 0 | 0 | 1 | 1 | 0 | |
| Li2024 | 0 | 0 | 0 | 0 | 0 | 0 | 0 | 0 | 0 | 0 | 0 | 0 | 0 | 0 | 0 | 0 | 0 | 0 | 0 | 0 | 0 | 0 | 0 | 0 | 0 | 0 | 0 | 0 | 0 | 0 | 0 | 0 | 0 | 0 | 0 | 1 | |
| CXJC:Compound Xuanju Capsules;CC:Clomiphene Citrate;LE:letrozole;WM: Western Medicine;KYE-CHM:Traditional Chinese medicine for tonifying the kidney and enhancing Yang;OI-WM:Western medicine for inducing ovulation;TCM:Traditional Chinese medicine;ACU:Acupuncture;CWM:conventional Western medicine;CPM:Chinese patent medicines;CHD:Chinese herbal decoctions;RKAB-CH:Reinforcing kidney and activating blood Chinese herbal;LE:letrozole;BTR:Bushen Tiaojing Recipes ;OID: ovulation induction drugs;DKD:Dingkundan;OWM:Other Western medicines;JFP:Jinfeng pills ;KTC:Kuntai Capsules;EE:Ethinylestradiol;CPA:cyproterone acetate tablets;HMG:Urogonadotropin;IVF-ET: In Vitro Fertilization-embryo Transfer;CHM:Chinese herbal medicine;D:Decoction;G:Granules;BOE:before oocyte extraction;FE:fresh embyro;F:frozen;DT:days transfer;HT:hours transfer;CTCWM:combine traditional Chinese and western medicine;CM+WM(TCMCM):Chinese medicine + Western medicine（Traditional Chinese Medicine Cycle Method）;TCMCT:Traditional Chinese medicine cycle therapy;OIP:ovulation induction protocol | | | | | | | | | | | | | | | | | | | | | | | | | | | | | | | | | | | | | |
