# Supplementary material for: The efficacy and safety of combined traditional Chinese and Western medicine treatment for PCOS-related infertility: an umbrella meta-analysis
Source: Front Med (Lausanne). 2026 Aug 6;13:1877844. doi: 10.3389/fmed.2026.1877844 (PMC13490117; doi:10.3389/fmed.2026.1877844)
Supplement: Supplementary file 4 [file Table_4.docx]

| **Table S4 AMSTAR Rating Scale** | | | | | | | | | | | | |
| --- | --- | --- | --- | --- | --- | --- | --- | --- | --- | --- | --- | --- |
| **Study** | **A priori design provided** | **Duplicate study selection & data extraction** | **At least two electronic databases searched** | **Status of publication used as an inclusion criterion** | **List of included and excluded studies provided** | **Characteristics of included studies provided** | **Scientific quality of included studies assessed** | **Scientific quality of the included studies used appropriately to form conclusions（GRADE）** | **Appropriate methods to combine studies** | **Publication bias assessed** | **Conflict of interest included** | **Total AMSTAR Score** |
| ZHOU Kunyan2014 | 0 | 1 | 1 | 0 | 0 | 1 | 1 | 0 | 1 | 0 | 0 | 5 |
| Qianwen Ma2023 | 0 | 1 | 1 | 0 | 1 | 1 | 1 | 0 | 1 | 0 | 1 | 7 |
| DU Xiu2020 | 0 | 1 | 1 | 0 | 1 | 1 | 1 | 0 | 1 | 1 | 0 | 7 |
| ZHAO Shuai2022 | 0 | 1 | 1 | 0 | 1 | 1 | 1 | 0 | 1 | 0 | 0 | 6 |
| YANG Ting2022 | 0 | 1 | 1 | 0 | 1 | 1 | 1 | 0 | 1 | 1 | 0 | 7 |
| HUANG Shouqia2021 | 0 | 1 | 1 | 0 | 1 | 1 | 1 | 0 | 1 | 0 | 0 | 6 |
| XUE Zhu2022 | 0 | 1 | 1 | 0 | 1 | 1 | 1 | 0 | 1 | 1 | 0 | 7 |
| XU Lifang2018 | 0 | 0 | 1 | 0 | 0 | 0 | 1 | 0 | 1 | 1 | 0 | 4 |
| SHENG Nan2021 | 0 | 1 | 1 | 0 | 1 | 1 | 1 | 0 | 1 | 1 | 0 | 7 |
| CHEN Jing2021 | 0 | 1 | 1 | 0 | 1 | 1 | 1 | 1 | 1 | 1 | 1 | 9 |
| DING Ke-fan2024 | 0 | 1 | 1 | 0 | 1 | 1 | 1 | 0 | 1 | 1 | 0 | 7 |
| YuXu2022 | 0 | 1 | 1 | 0 | 1 | 1 | 1 | 0 | 1 | 1 | 1 | 8 |
| LIU Ying2019 | 0 | 1 | 1 | 0 | 0 | 1 | 1 | 0 | 1 | 0 | 0 | 5 |
| Chang Liu2021 | 0 | 1 | 1 | 0 | 1 | 1 | 1 | 0 | 1 | 1 | 1 | 8 |
| CAI Shu2016 | 0 | 0 | 1 | 0 | 0 | 1 | 1 | 0 | 1 | 1 | 0 | 5 |
| WU Haiyan2019 | 0 | 0 | 1 | 0 | 1 | 1 | 1 | 0 | 1 | 1 | 0 | 6 |
| LI Huimin2024 | 1 | 1 | 1 | 0 | 1 | 1 | 1 | 0 | 1 | 1 | 0 | 8 |

CC:Clomiphene Citrate;LE:letrozole;HCG:Human Chorionic Gonadotropin;HMG:Urogonadotropin;CHM:Chinese Herbal Medicine;IVF-ET:In Vitro Fertilization-embryo Transfer;T:Testosterone;BMI:Body Mass Index;LH:Luteinizing Hormone;FSH:Follicle-stimulating Hormone;E2:Estradiol;BBT:Basal Body Temperature;HGF:Hepatocyte Growth Factor;VEGF:Vascular Endothelial Growth Facto;TNF-α:Tumor Necrosis Factor-α;OHSS:Ovarian Hyper-stimulation Syndrome
