# Supplementary material for: The efficacy and safety of combined traditional Chinese and Western medicine treatment for PCOS-related infertility: an umbrella meta-analysis
Source: Front Med (Lausanne). 2026 Aug 6;13:1877844. doi: 10.3389/fmed.2026.1877844 (PMC13490117; doi:10.3389/fmed.2026.1877844)
Supplement: Supplementary file 5 [file Table_5.docx]

| **Table S5 GRADE Rating** | | | | | | | | | | | | | |
| --- | --- | --- | --- | --- | --- | --- | --- | --- | --- | --- | --- | --- | --- |
| **Study** | **Intervention** | **Diseases** | **outcome** | **No. of studies(T/R)** | **Risk of Bias** | **Inconsistency** | **Indirectness** | **Imprecision** | **Publication bias** | **Plausible confounding** | **Magnitude of effect** | **Dose-response gradient** | **Quality** |
| ZHOU Kunyan2014 | Compound Xuanju Capsules +CC | Polycystic ovary syndrome combined with infertility | the pregnancy rate | 3/3 | no serious | no serious | no serious | serious | serious | would not | no | no | moderate |
| ZHOU Kunyan2014 | Compound Xuanju Capsules +CC | Polycystic ovary syndrome combined with infertility | Ovulation rate during the cycle | 1/1 | no serious | na | no serious | serious | serious | would not | no | no | moderate |
| ZHOU Kunyan2014 | Compound Xuanju Capsules +CC | Polycystic ovary syndrome combined with infertility | Menstrual improvement rate | 3/3 | no serious | no serious | no serious | serious | serious | would not | yes | no | moderate |
| ZHOU Kunyan2014 | Compound Xuanju Capsules +CC | Polycystic ovary syndrome combined with infertility | T | 1/1 | no serious | na | no serious | serious | serious | would not | no | no | moderate |
| ZHOU Kunyan2014 | Compound Xuanju Capsules +LE | Polycystic ovary syndrome combined with infertility | the pregnancy rate | 1/1 | no serious | na | no serious | serious | serious | would not | no | no | moderate |
| ZHOU Kunyan2014 | Compound Xuanju Capsules +LE | Polycystic ovary syndrome combined with infertility | Ovulation rate during the cycle | 1/1 | no serious | na | no serious | serious | serious | would not | no | no | moderate |
| ZHOU Kunyan2014 | Compound Xuanju Capsules +LE | Polycystic ovary syndrome combined with infertility | Menstrual improvement rate | 1/1 | no serious | na | no serious | serious | serious | would not | no | no | moderate |
| ZHOU Kunyan2014 | Compound Xuanju Capsules +LE | Polycystic ovary syndrome combined with infertility | BMI | 1/1 | no serious | na | no serious | serious | serious | would not | no | no | moderate |
| Qianwen Ma2023 | Compound Xuanju Capsules + Hormone | Polycystic ovary syndrome combined with infertility | the ovulation rate | 6/6 | no serious | no serious | no serious | serious | na | would not | yes | no | high |
| Qianwen Ma2023 | Compound Xuanju Capsules + Hormone | Polycystic ovary syndrome combined with infertility | the pregnancy rate | 11/11 | no serious | no serious | no serious | serious | serious | would not | yes | no | moderate |
| Qianwen Ma2023 | Compound Xuanju Capsules + Hormone | Polycystic ovary syndrome combined with infertility | LH level | 12/12 | no serious | serious | no serious | serious | serious | would not | yes | no | low |
| Qianwen Ma2023 | Compound Xuanju Capsules + Hormone | Polycystic ovary syndrome combined with infertility | FSH level | 12/12 | no serious | serious | no serious | serious | serious | would not | no | no | low |
| Qianwen Ma2023 | Compound Xuanju Capsules + Hormone | Polycystic ovary syndrome combined with infertility | E2 leve | 7/7 | no serious | serious | no serious | serious | serious | would not | no | no | low |
| Qianwen Ma2023 | Compound Xuanju Capsules + Hormone | Polycystic ovary syndrome combined with infertility | LH/FSH ratio | 2/2 | no serious | serious | no serious | serious | serious | would not | no | no | low |
| Qianwen Ma2023 | Compound Xuanju Capsules + Hormone | Polycystic ovary syndrome combined with infertility | T leve | 9/9 | no serious | serious | no serious | serious | na | would not | no | no | moderate |
| Qianwen Ma2023 | Compound Xuanju Capsules + Hormone | Polycystic ovary syndrome combined with infertility | endometrial thickness | 5/5 | no serious | serious | no serious | serious | serious | would not | no | no | low |
| Qianwen Ma2023 | Compound Xuanju Capsules + Hormone | Polycystic ovary syndrome combined with infertility | overall rate of effective treatment | 6/6 | no serious | no serious | no serious | serious | serious | would not | yes | no | moderate |
| Qianwen Ma2023 | Compound Xuanju Capsules + Hormone | Polycystic ovary syndrome combined with infertility | overall rate of effective Chinese medicine-based treatment | 2/2 | no serious | no serious | no serious | serious | serious | would not | yes | no | moderate |
| Qianwen Ma2023 | Compound Xuanju Capsules + Hormone | Polycystic ovary syndrome combined with infertility | BBT | 2/2 | no serious | no serious | no serious | serious | na | would not | yes | no | high |
| Qianwen Ma2023 | Compound Xuanju Capsules + Hormone | Polycystic ovary syndrome combined with infertility | left antral follicle count | 2/2 | no serious | no serious | no serious | serious | na | would not | no | no | high |
| Qianwen Ma2023 | Compound Xuanju Capsules + Hormone | Polycystic ovary syndrome combined with infertility | right antral follicle count | 2/2 | no serious | no serious | no serious | serious | na | would not | no | no | high |
| Qianwen Ma2023 | Compound Xuanju Capsules + Hormone | Polycystic ovary syndrome combined with infertility | left ovarian volume | 2/2 | no serious | no serious | no serious | serious | na | would not | no | no | high |
| Qianwen Ma2023 | Compound Xuanju Capsules + Hormone | Polycystic ovary syndrome combined with infertility | right ovarian volume | 2/2 | no serious | no serious | no serious | serious | na | would not | no | no | high |
| Qianwen Ma2023 | Compound Xuanju Capsules + Hormone | Polycystic ovary syndrome combined with infertility | follicle count | 2/2 | no serious | no serious | no serious | serious | na | would not | no | no | high |
| Qianwen Ma2023 | Compound Xuanju Capsules + Hormone | Polycystic ovary syndrome combined with infertility | maximum follicle diamete | 2/2 | no serious | serious | no serious | serious | na | would not | no | no | moderate |
| Qianwen Ma2023 | Compound Xuanju Capsules + Hormone | Polycystic ovary syndrome combined with infertility | HGF | 2/2 | no serious | serious | no serious | serious | na | would not | no | no | moderate |
| Qianwen Ma2023 |  | Polycystic ovary syndrome combined with infertility | VEGF | 2/2 | no serious | no serious | no serious | serious | na | would not | no | no | high |
| DU Xiu2020 | Compound Xuanju Capsules + Western Medicine | Polycystic ovary syndrome combined with infertility | the pregnancy rate | 14/14 | no serious | no serious | no serious | serious | no serious | would not | no | no | high |
| DU Xiu2020 | Compound Xuanju Capsules + Western Medicine | Polycystic ovary syndrome combined with infertility | Ovulation rate during the cycle | 6/6 | no serious | serious | no serious | serious | serious | would not | no | no | low |
| DU Xiu2020 | Compound Xuanju Capsules + Western Medicine | Polycystic ovary syndrome combined with infertility | Comparison of the improvement of TCM symptoms | 6/6 | no serious | serious | no serious | serious | serious | would not | no | no | low |
| DU Xiu2020 | Compound Xuanju Capsules + Western Medicine | Polycystic ovary syndrome combined with infertility | T | 5/5 | no serious | serious | no serious | serious | serious | would not | no | no | low |
| DU Xiu2020 | Compound Xuanju Capsules + Western Medicine | Polycystic ovary syndrome combined with infertility | endometrial thickness | 6/6 | no serious | serious | no serious | serious | serious | would not | no | no | low |
| ZHAO Shuai2022 | Traditional Chinese medicine for tonifying the kidney and enhancing Yang + Western medicine for inducing ovulation | Polycystic ovary syndrome combined with infertility | total effective rate | 7/7/ | no serious | no serious | no serious | serious | na | would not | no | no | high |
| ZHAO Shuai2022 | Traditional Chinese medicine for tonifying the kidney and enhancing Yang + Western medicine for inducing ovulation | Polycystic ovary syndrome combined with infertility | the pregnancy rate | 14/14 | no serious | no serious | no serious | serious | serious | would not | no | no | moderate |
| ZHAO Shuai2022 | Traditional Chinese medicine for tonifying the kidney and enhancing Yang + Western medicine for inducing ovulation | Polycystic ovary syndrome combined with infertility | luteinizing hormone | 11/11 | no serious | serious | no serious | serious | na | would not | no | no | moderate |
| ZHAO Shuai2022 | Traditional Chinese medicine for tonifying the kidney and enhancing Yang + Western medicine for inducing ovulation | Polycystic ovary syndrome combined with infertility | follicle-stimulating hormone | 10/10 | no serious | serious | no serious | serious | na | would not | no | no | moderate |
| ZHAO Shuai2022 | Traditional Chinese medicine for tonifying the kidney and enhancing Yang + Western medicine for inducing ovulation | Polycystic ovary syndrome combined with infertility | luteinizing hormone/follicle-stimulating hormone | 5/5 | no serious | serious | no serious | serious | na | would not | no | no | moderate |
| ZHAO Shuai2022 | Traditional Chinese medicine for tonifying the kidney and enhancing Yang + Western medicine for inducing ovulation | Polycystic ovary syndrome combined with infertility | estradiol | 8/8 | no serious | serious | no serious | serious | na | would not | no | no | moderate |
| ZHAO Shuai2022 | Traditional Chinese medicine for tonifying the kidney and enhancing Yang + Western medicine for inducing ovulation | Polycystic ovary syndrome combined with infertility | T | 8/8 | no serious | serious | no serious | serious | na | would not | no | no | moderate |
| ZHAO Shuai2022 | Traditional Chinese medicine for tonifying the kidney and enhancing Yang + Western medicine for inducing ovulation | Polycystic ovary syndrome combined with infertility | endometrial thickness | 8/8 | no serious | no serious | no serious | serious | na | would not | no | no | high |
| ZHAO Shuai2022 | Traditional Chinese medicine for tonifying the kidney and enhancing Yang + Western medicine for inducing ovulation | Polycystic ovary syndrome combined with infertility | endometrial spiral artery pulsatility index | 2/2 | no serious | no serious | no serious | serious | na | would not | no | no | high |
| ZHAO Shuai2022 | Traditional Chinese medicine for tonifying the kidney and enhancing Yang + Western medicine for inducing ovulation | Polycystic ovary syndrome combined with infertility | endometrial spiral artery resistance index | 3/3 | no serious | no serious | no serious | serious | na | would not | no | no | high |
| YANG Ting2022 | Traditional Chinese medicine/(traditional Chinese medicine + acupuncture) + conventional Western medicine | Polycystic ovary syndrome combined with infertility | Comprehensive clinical efficacy | 14/14 | no serious | no serious | no serious | serious | no serious | would not | yes | no | high |
| YANG Ting2022 | Traditional Chinese medicine/(traditional Chinese medicine + acupuncture) + conventional Western medicine | Polycystic ovary syndrome combined with infertility | the pregnancy rate | 32/32 | no serious | no serious | no serious | serious | no serious | would not | yes | no | high |
| YANG Ting2022 | Traditional Chinese medicine/(traditional Chinese medicine + acupuncture) + conventional Western medicine | Polycystic ovary syndrome combined with infertility | Bad rectification rate | 10/10 | no serious | no serious | no serious | serious | no serious | would not | no | no | high |
| YANG Ting2022 | Traditional Chinese medicine/(traditional Chinese medicine + acupuncture) + conventional Western medicine | Polycystic ovary syndrome combined with infertility | the abortion rate | 7/7 | no serious | no serious | no serious | serious | serious | would not | yes | no | moderate |
| YANG Ting2022 | Traditional Chinese medicine/(traditional Chinese medicine + acupuncture) + conventional Western medicine | Polycystic ovary syndrome combined with infertility | the ovulation rate | 11/11 | no serious | no serious | no serious | serious | serious | would not | yes | no | moderate |
| YANG Ting2022 | Traditional Chinese medicine/(traditional Chinese medicine + acupuncture) + conventional Western medicine | Polycystic ovary syndrome combined with infertility | LH level | 13/13 | no serious | no serious | no serious | serious | serious | would not | no | no | moderate |
| YANG Ting2022 | Traditional Chinese medicine/(traditional Chinese medicine + acupuncture) + conventional Western medicine | Polycystic ovary syndrome combined with infertility | LH/FSH | 4/4 | no serious | no serious | no serious | serious | serious | would not | no | no | moderate |
| YANG Ting2022 | Traditional Chinese medicine/(traditional Chinese medicine + acupuncture) + conventional Western medicine | Polycystic ovary syndrome combined with infertility | Tlevel | 9/9 | no serious | no serious | no serious | serious | serious | would not | no | no | moderate |
| YANG Ting2022 | Traditional Chinese medicine/(traditional Chinese medicine + acupuncture) + conventional Western medicine | Polycystic ovary syndrome combined with infertility | endometrial thickness | 10/10 | no serious | serious | no serious | serious | no serious | would not | no | no | moderate |
| YANG Ting2022 | Traditional Chinese medicine/(traditional Chinese medicine + acupuncture) + conventional Western medicine | Polycystic ovary syndrome combined with infertility | endometrial spiral artery pulsatility index | 3/3 | no serious | no serious | no serious | serious | serious | would not | no | no | moderate |
| YANG Ting2022 | Traditional Chinese medicine/(traditional Chinese medicine + acupuncture) + conventional Western medicine | Polycystic ovary syndrome combined with infertility | endometrial spiral artery resistance index | 3/3 | no serious | no serious | no serious | serious | na | would not | no | no | high |
| YANG Ting2022 | Traditional Chinese medicine/(traditional Chinese medicine + acupuncture) + conventional Western medicine | Polycystic ovary syndrome combined with infertility | TNF-α | 2/2 | no serious | no serious | no serious | serious | serious | would not | no | no | moderate |
| HUANG Shouqia2021 | Acupuncture + Western medicine | Polycystic ovary syndrome combined with infertility | the pregnancy rate | 14/14 | no serious | no serious | no serious | serious | na | would not | no | no | high |
| HUANG Shouqia2021 | Acupuncture + Western medicine | Polycystic ovary syndrome combined with infertility | Ovulation rate during the cycle | 4/4 | no serious | no serious | no serious | serious | na | would not | no | no | high |
| HUANG Shouqia2021 | Acupuncture + Western medicine | Polycystic ovary syndrome combined with infertility | the ovulation rate | 8/8 | no serious | no serious | no serious | serious | na | would not | no | no | high |
| HUANG Shouqia2021 | Acupuncture + Western medicine | Polycystic ovary syndrome combined with infertility | FSH | 7/7 | no serious | serious | no serious | serious | na | would not | no | no | moderate |
| HUANG Shouqia2021 | Acupuncture + Western medicine | Polycystic ovary syndrome combined with infertility | LH | 7/7 | no serious | serious | no serious | serious | na | would not | no | no | moderate |
| HUANG Shouqia2021 | Acupuncture + Western medicine | Polycystic ovary syndrome combined with infertility | LH/FSH | 3/3 | no serious | serious | no serious | serious | na | would not | no | no | moderate |
| HUANG Shouqia2021 | Acupuncture + Western medicine | Polycystic ovary syndrome combined with infertility | T | 8/8 | no serious | serious | no serious | serious | na | would not | no | no | moderate |
| XUE Zhu2022 | Chinese patent medicines/Chinese herbal decoctions + Western medicines | Polycystic ovary syndrome combined with infertility | Total clinical effective rate | 13/13 | no serious | no serious | no serious | serious | no serious | would not | yes | no | high |
| XUE Zhu2022 | Chinese patent medicine + Western medicine | Polycystic ovary syndrome combined with infertility | Total clinical effective rate | 3/3 | no serious | no serious | no serious | serious | no serious | would not | yes | no | high |
| XUE Zhu2022 | Chinese herbal decoctions + Western medicine | Polycystic ovary syndrome combined with infertility | Total clinical effective rate | 10/10 | no serious | no serious | no serious | serious | no serious | would not | yes | no | high |
| XUE Zhu2022 | Chinese patent medicines/Chinese herbal decoctions + Western medicines | Polycystic ovary syndrome combined with infertility | the ovulation rate | 7/7 | no serious | no serious | no serious | serious | na | would not | yes | no | high |
| XUE Zhu2022 | Chinese patent medicines/Chinese herbal decoctions + Western medicines | Polycystic ovary syndrome combined with infertility | the pregnancy rate | 16/16 | no serious | no serious | no serious | serious | no serious | would not | yes | no | high |
| XUE Zhu2022 | Chinese patent medicine + Western medicine | Polycystic ovary syndrome combined with infertility | the pregnancy rate | 4/4 | no serious | no serious | no serious | serious | no serious | would not | yes | no | high |
| XUE Zhu2022 | Chinese herbal decoctions + Western medicine | Polycystic ovary syndrome combined with infertility | the pregnancy rate | 12/12 | no serious | no serious | no serious | serious | no serious | would not | yes | no | high |
| XUE Zhu2022 | Chinese patent medicines/Chinese herbal decoctions + Western medicines | Polycystic ovary syndrome combined with infertility | LH | 15/15 | no serious | serious | no serious | serious | no serious | would not | no | no | moderate |
| XUE Zhu2022 | Chinese patent medicines/Chinese herbal decoctions + Western medicines | Polycystic ovary syndrome combined with infertility | FSH | 13/13 | no serious | serious | no serious | serious | no serious | would not | no | no | moderate |
| XUE Zhu2022 | Chinese patent medicines/Chinese herbal decoctions + Western medicines | Polycystic ovary syndrome combined with infertility | LH/FSH | 6/6 | no serious | serious | no serious | serious | na | would not | no | no | moderate |
| XUE Zhu2022 | Chinese patent medicines/Chinese herbal decoctions + Western medicines | Polycystic ovary syndrome combined with infertility | T | 14/14 | no serious | serious | no serious | serious | no serious | would not | no | no | moderate |
| XUE Zhu2022 | Chinese patent medicines/Chinese herbal decoctions + Western medicines | Polycystic ovary syndrome combined with infertility | endometrial thickness | 8/8 | no serious | serious | no serious | serious | na | would not | no | no | moderate |
| XUE Zhu2022 | Chinese patent medicines/Chinese herbal decoctions + Western medicines | Polycystic ovary syndrome combined with infertility | The rate of spontaneous abortion | 2/2 | no serious | no serious | no serious | serious | na | would not | no | no | high |
| XU Lifang2018 | Reinforcing kidney and activating blood Chinese herbal +Western medicine | Polycystic ovary syndrome combined with infertility | total effective rate | 18/18 | no serious | no serious | no serious | serious | serious | would not | yes | no | moderate |
| XU Lifang2018 | Reinforcing kidney and activating blood Chinese herbal +Western medicine | Polycystic ovary syndrome combined with infertility | the pregnancy rate | 18/18 | no serious | no serious | no serious | serious | serious | would not | yes | no | moderate |
| XU Lifang2018 | Reinforcing kidney and activating blood Chinese herbal +Western medicine | Polycystic ovary syndrome combined with infertility | security analysis | 10/10 | no serious | serious | no serious | serious | serious | would not | yes | no | low |
| SHENG Nan2021 | Reinforcing kidney and activating blood Chinese herbal+LE | Polycystic ovary syndrome combined with infertility | total effective rate | 8/8 | no serious | no serious | no serious | serious | serious | would not | yes | no | moderate |
| SHENG Nan2021 | Reinforcing kidney and activating blood Chinese herbal+LE | Polycystic ovary syndrome combined with infertility | the pregnancy rate | 8/8 | no serious | no serious | no serious | serious | serious | would not | yes | no | moderate |
| SHENG Nan2021 | Reinforcing kidney and activating blood Chinese herbal+LE | Polycystic ovary syndrome combined with infertility | endometrial thickness | 6/6 | no serious | serious | no serious | serious | serious | would not | no | no | low |
| SHENG Nan2021 | Reinforcing kidney and activating blood Chinese herbal+LE | Polycystic ovary syndrome combined with infertility | LH | 5/5 | no serious | serious | no serious | serious | serious | would not | no | no | low |
| SHENG Nan2021 | Reinforcing kidney and activating blood Chinese herbal+LE | Polycystic ovary syndrome combined with infertility | FSH | 3/3 | no serious | no serious | no serious | serious | serious | would not | no | no | moderate |
| CHEN Jing2021 | Bushen Tiaojing Recipes + ovulation induction drugs | Polycystic ovary syndrome combined with infertility | the ovulation rate | 14/14 | no serious | no serious | no serious | serious | serious | would not | no | no | moderate |
| CHEN Jing2021 | Bushen Tiaojing Recipes + ovulation induction drugs | Polycystic ovary syndrome combined with infertility | the pregnancy rate | 19/19 | no serious | no serious | no serious | serious | serious | would not | no | no | moderate |
| CHEN Jing2021 | Bushen Tiaojing Recipes + ovulation induction drugs | Polycystic ovary syndrome combined with infertility | Incidence of adverse reactions | 5/5 | no serious | no serious | no serious | serious | na | would not | yes | no | high |
| CHEN Jing2021 | Bushen Tiaojing Recipes + ovulation induction drugs | Polycystic ovary syndrome combined with infertility | T | 16/16 | no serious | serious | no serious | serious | na | would not | no | no | moderate |
| CHEN Jing2021 | Bushen Tiaojing Recipes + ovulation induction drugs | Polycystic ovary syndrome combined with infertility | LH | 17/17 | no serious | serious | no serious | serious | na | would not | no | no | moderate |
| CHEN Jing2021 | Bushen Tiaojing Recipes + ovulation induction drugs | Polycystic ovary syndrome combined with infertility | FSH | 16/16 | no serious | serious | no serious | serious | na | would not | no | no | moderate |
| CHEN Jing2021 | Bushen Tiaojing Recipes + ovulation induction drugs | Polycystic ovary syndrome combined with infertility | estradiol | 15/15 | no serious | serious | no serious | serious | na | would not | no | no | moderate |
| CHEN Jing2021 | Bushen Tiaojing Recipes + ovulation induction drugs | Polycystic ovary syndrome combined with infertility | endometrial thickness | 13/13 | no serious | serious | no serious | serious | na | would not | no | no | moderate |
| CHEN Jing2021 | Bushen Tiaojing Recipes + ovulation induction drugs | Polycystic ovary syndrome combined with infertility | ovarian volume | 5/5 | no serious | serious | no serious | serious | na | would not | no | no | moderate |
| DING Ke-fan2024 | Dingkundan+LE | Polycystic ovary syndrome combined with infertility | endometrial thickness | 8/8 | no serious | serious | no serious | serious | no serious | would not | no | no | moderate |
| DING Ke-fan2024 | Dingkundan+LE | Polycystic ovary syndrome combined with infertility | the ovulation rate | 6/6 | no serious | serious | no serious | serious | serious | would not | no | no | low |
| DING Ke-fan2024 | Dingkundan+LE | Polycystic ovary syndrome combined with infertility | the pregnancy rate | 9/9 | no serious | no serious | no serious | serious | serious | would not | no | no | moderate |
| DING Ke-fan2024 | Dingkundan+Other Western medicines | Polycystic ovary syndrome combined with infertility | endometrial thickness | 4/4 | no serious | serious | no serious | serious | no serious | would not | no | no | moderate |
| DING Ke-fan2024 | Dingkundan+Other Western medicines | Polycystic ovary syndrome combined with infertility | the ovulation rate | 6/6 | no serious | no serious | no serious | serious | serious | would not | no | no | moderate |
| DING Ke-fan2024 | Dingkundan+Other Western medicines | Polycystic ovary syndrome combined with infertility | the pregnancy rate | 7/7 | no serious | no serious | no serious | serious | serious | would not | no | no | moderate |
| DING Ke-fan2024 | Dingkundan+Western medicine | Polycystic ovary syndrome combined with infertility | endometrial thickness | 12/12 | no serious | serious | no serious | serious | no serious | would not | no | no | moderate |
| DING Ke-fan2024 | Dingkundan+Western medicine | Polycystic ovary syndrome combined with infertility | the number of mature follicles | 10/10 | no serious | serious | no serious | serious | no serious | would not | no | no | moderate |
| DING Ke-fan2024 | Dingkundan+Western medicine | Polycystic ovary syndrome combined with infertility | the ovulation rate | 12/12 | no serious | no serious | no serious | serious | serious | would not | no | no | moderate |
| DING Ke-fan2024 | Dingkundan+Western medicine | Polycystic ovary syndrome combined with infertility | the pregnancy rate | 16/16 | no serious | no serious | no serious | serious | serious | would not | no | no | moderate |
| DING Ke-fan2024 | Dingkundan+Western medicine | Polycystic ovary syndrome combined with infertility | LH | 12/12 | no serious | serious | no serious | serious | serious | would not | no | no | low |
| DING Ke-fan2024 | Dingkundan+Western medicine | Polycystic ovary syndrome combined with infertility | total effective rate | 6/6 | no serious | no serious | no serious | serious | serious | would not | no | no | moderate |
| YuXu2022 | Jinfeng pills + Western medicine | Polycystic ovary syndrome combined with infertility | the effective rate | 6/6 | no serious | no serious | no serious | serious | no serious | would not | no | no | high |
| YuXu2022 | Jinfeng pills + Western medicine | Polycystic ovary syndrome combined with infertility | FSH | 4/4 | no serious | serious | no serious | serious | no serious | would not | no | no | moderate |
| YuXu2022 | Jinfeng pills + Western medicine | Polycystic ovary syndrome combined with infertility | LH | 4/4 | no serious | serious | no serious | serious | no serious | would not | no | no | moderate |
| YuXu2022 | Jinfeng pills + Western medicine | Polycystic ovary syndrome combined with infertility | E2 | 4/4 | no serious | serious | no serious | serious | no serious | would not | no | no | moderate |
| YuXu2022 | Jinfeng pills + Western medicine | Polycystic ovary syndrome combined with infertility | T | 4/4 | no serious | serious | no serious | serious | no serious | would not | no | no | moderate |
| YuXu2022 | Jinfeng pills + Western medicine | Polycystic ovary syndrome combined with infertility | The ovulation rate | 3/3 | no serious | no serious | no serious | serious | no serious | would not | no | no | high |
| YuXu2022 | Jinfeng pills + Western medicine | Polycystic ovary syndrome combined with infertility | The pregnancy rate | 3/3 | no serious | no serious | no serious | serious | no serious | would not | no | no | high |
| LIU Ying2019 | Kuntai Capsules+CC | Polycystic ovary syndrome combined with infertility | The pregnancy rate | 2/2 | no serious | no serious | no serious | serious | serious | would not | no | no | moderate |
| LIU Ying2019 | Kuntai Capsules+CC | Polycystic ovary syndrome combined with infertility | The ovulation rate | 2/2 | no serious | serious | no serious | serious | serious | would not | no | no | low |
| LIU Ying2019 | Kuntai Capsules+CC+HCG | Polycystic ovary syndrome combined with infertility | The pregnancy rate | 1/1 | no serious | na | no serious | serious | serious | would not | no | no | moderate |
| LIU Ying2019 | Kuntai Capsules+CC+HCG | Polycystic ovary syndrome combined with infertility | The ovulation rate | 1/1 | no serious | na | no serious | serious | serious | would not | no | no | moderate |
| LIU Ying2019 | Kuntai Capsules+Ethinylestradiol and cyproterone acetate tablets+CC+HMG | Polycystic ovary syndrome combined with infertility | The pregnancy rate | 2/2 | no serious | no serious | no serious | serious | serious | would not | no | no | moderate |
| LIU Ying2019 | Kuntai Capsules+Ethinylestradiol and cyproterone acetate tablets+CC+HMG | Polycystic ovary syndrome combined with infertility | The ovulation rate | 2/2 | no serious | no serious | no serious | serious | serious | would not | no | no | moderate |
| LIU Ying2019 | Kuntai Capsules+LE | Polycystic ovary syndrome combined with infertility | The pregnancy rate | 3/3 | no serious | no serious | no serious | serious | serious | would not | yes | no | moderate |
| LIU Ying2019 | Kuntai Capsules+LE | Polycystic ovary syndrome combined with infertility | The ovulation rate | 1/1 | no serious | na | no serious | serious | serious | would not | no | no | moderate |
| LIU Ying2019 | Kuntai Capsules+Ethinylestradiol and cyproterone acetate tablets | Polycystic ovary syndrome combined with infertility | The pregnancy rate | 3/3 | no serious | serious | no serious | serious | serious | would not | no | no | low |
| LIU Ying2019 | Kuntai Capsules+Ethinylestradiol and cyproterone acetate tablets | Polycystic ovary syndrome combined with infertility | The ovulation rate | 2/2 | no serious | no serious | no serious | serious | serious | would not | no | no | moderate |
| LIU Ying2019 | Kuntai Capsules+Western medicine | Polycystic ovary syndrome combined with infertility | The pregnancy rate | 11/11 | no serious | no serious | no serious | serious | serious | would not | no | no | moderate |
| LIU Ying2019 | Kuntai Capsules+Western medicine | Polycystic ovary syndrome combined with infertility | The ovulation rate | 8/8 | no serious | no serious | no serious | serious | serious | would not | no | no | moderate |
| Chang Liu2021 | a combination of IVF-ET and CHM | Polycystic ovary syndrome combined with infertility | the clinical pregnancy rate | 10/10 | no serious | no serious | no serious | serious | serious | would not | yes | no | moderate |
| Chang Liu2021 | a combination of IVF-ET and CHM | Polycystic ovary syndrome combined with infertility | he abortion rate | 4/4 | no serious | no serious | no serious | serious | serious | would not | no | no | moderate |
| Chang Liu2021 | a combination of IVF-ET and CHM | Polycystic ovary syndrome combined with infertility | the OHSS incidence. | 9/9 | no serious | no serious | no serious | serious | serious | would not | yes | no | moderate |
| Chang Liu2021 | a combination of IVF-ET and CHM(Decoction) | Polycystic ovary syndrome combined with infertility | the clinical pregnancy rate | 6/6 | no serious | no serious | no serious | serious | serious | would not | yes | no | moderate |
| Chang Liu2021 | a combination of IVF-ET and CHM(Granules) | Polycystic ovary syndrome combined with infertility | the clinical pregnancy rate | 3/3 | no serious | no serious | no serious | serious | serious | would not | yes | no | moderate |
| Chang Liu2021 | a combination of IVF-ET and CHM((Decoction+Granules) | Polycystic ovary syndrome combined with infertility | the clinical pregnancy rate | 9/9 | no serious | no serious | no serious | serious | serious | would not | yes | no | moderate |
| Chang Liu2021 | a combination of IVF-ET and CHM(before oocyte extraction) | Polycystic ovary syndrome combined with infertility | the clinical pregnancy rate | 7/7 | no serious | no serious | no serious | serious | serious | would not | yes | no | moderate |
| Chang Liu2021 | a combination of IVF-ET and CHM(over all the process of IVF) | Polycystic ovary syndrome combined with infertility | the clinical pregnancy rate | 2/2 | no serious | no serious | no serious | serious | serious | would not | no | no | moderate |
| Chang Liu2021 | a combination of IVF-ET(frozen embyro） and CHM | Polycystic ovary syndrome combined with infertility | the clinical pregnancy rate | 2/2 | no serious | no serious | no serious | serious | serious | would not | no | no | moderate |
| Chang Liu2021 | a combination of IVF-ET(fresh embyro） and CHM | Polycystic ovary syndrome combined with infertility | the clinical pregnancy rate | 8/8 | no serious | no serious | no serious | serious | serious | would not | yes | no | moderate |
| Chang Liu2021 | a combination of IVF-ET（frozen and fresh embyro） and CHM | Polycystic ovary syndrome combined with infertility | the clinical pregnancy rate | 10/10 | no serious | no serious | no serious | serious | serious | would not | yes | no | moderate |
| Chang Liu2021 | a combination of IVF-ET(fresh embryo, 2-3 days transfer） and CHM | Polycystic ovary syndrome combined with infertility | the clinical pregnancy rate | 4/4 | no serious | no serious | no serious | serious | serious | would not | yes | no | moderate |
| Chang Liu2021 | a combination of IVF-ET(fresh embryo, 18-22 hours transfer） and CHM | Polycystic ovary syndrome combined with infertility | the clinical pregnancy rate | 2/2 | no serious | no serious | no serious | serious | serious | would not | yes | no | moderate |
| Chang Liu2021 | a combination of IVF-ET(fresh embryo, 2-3 days transfer and fresh embryo, 18-22 hours transfer） and CHM | Polycystic ovary syndrome combined with infertility | the clinical pregnancy rate | 6/6 | no serious | no serious | no serious | serious | serious | would not | yes | no | moderate |
| CAI Shu2016 | combine traditional Chinese and western medicine | Polycystic ovary syndrome combined with infertility | The ovulation rate | 11/11 | no serious | serious | no serious | serious | no serious | would not | yes | no | moderate |
| CAI Shu2016 | combine traditional Chinese and western medicine | Polycystic ovary syndrome combined with infertility | The pregnancy rate | 13/13 | no serious | no serious | no serious | serious | no serious | would not | yes | no | high |
| CAI Shu2016 | combine traditional Chinese and western medicine | Polycystic ovary syndrome combined with infertility | effective rate | 6/6 | no serious | no serious | no serious | serious | no serious | would not | yes | no | high |
| WU Haiyan2019 | Chinese medicine + Western medicine（Traditional Chinese Medicine Cycle Method） | Polycystic ovary syndrome combined with infertility | Total clinical efficiency | 58/58 | no serious | no serious | no serious | serious | serious | would not | yes | no | moderate |
| WU Haiyan2019 | Chinese medicine + Western medicine（Traditional Chinese Medicine Cycle Method） | Polycystic ovary syndrome combined with infertility | The ovulation rate | 26/26 | no serious | serious | no serious | serious | na | would not | yes | no | moderate |
| WU Haiyan2019 | Chinese medicine + Western medicine（Traditional Chinese Medicine Cycle Method） | Polycystic ovary syndrome combined with infertility | The pregnancy rate | 51/51 | no serious | no serious | no serious | serious | na | would not | yes | no | high |
| WU Haiyan2019 | acupuncture + Western medicine（Traditional Chinese Medicine Cycle Method） | Polycystic ovary syndrome combined with infertility | Total clinical efficiency | 19/19 | no serious | no serious | no serious | serious | serious | would not | yes | no | moderate |
| WU Haiyan2019 | acupuncture + Western medicine（Traditional Chinese Medicine Cycle Method） | Polycystic ovary syndrome combined with infertility | The ovulation rate | 11/11 | no serious | no serious | no serious | serious | na | would not | yes | no | high |
| WU Haiyan2019 | acupuncture + Western medicine（Traditional Chinese Medicine Cycle Method） | Polycystic ovary syndrome combined with infertility | The pregnancy rate | 16/16 | no serious | no serious | no serious | serious | na | would not | yes | no | high |
| LI Huimin2024 | Traditional Chinese medicine cycle therapy + ovulation induction protocol | Polycystic ovary syndrome combined with infertility | The pregnancy rate | 15/15 | no serious | no serious | no serious | serious | no serious | would not | no | no | high |
| LI Huimin2024 | Traditional Chinese medicine cycle therapy + ovulation induction protocol | Polycystic ovary syndrome combined with infertility | total effective rate | 8/8 | no serious | no serious | no serious | serious | na | would not | no | no | high |
| LI Huimin2024 | Traditional Chinese medicine cycle therapy + ovulation induction protocol | Polycystic ovary syndrome combined with infertility | The ovulation rate | 11/11 | no serious | no serious | no serious | serious | na | would not | no | no | high |
| LI Huimin2024 | Traditional Chinese medicine cycle therapy + ovulation induction protocol | Polycystic ovary syndrome combined with infertility | Androgen level | 6/6 | no serious | no serious | no serious | serious | na | would not | no | no | high |
| LI Huimin2024 | Traditional Chinese medicine cycle therapy + ovulation induction protocol | Polycystic ovary syndrome combined with infertility | LH | 6/6 | no serious | no serious | no serious | serious | na | would not | no | no | high |
| LI Huimin2024 | Traditional Chinese medicine cycle therapy + ovulation induction protocol | Polycystic ovary syndrome combined with infertility | endometrial thickness | 3/3 | no serious | no serious | no serious | serious | na | would not | no | no | high |
| LI Huimin2024 | Traditional Chinese medicine cycle therapy + ovulation induction protocol | Polycystic ovary syndrome combined with infertility | Incidence of OHSS | 3/3 | no serious | no serious | no serious | serious | na | would not | yes | no | high |
| LI Huimin2024 | Traditional Chinese medicine cycle therapy + ovulation induction protocol | Polycystic ovary syndrome combined with infertility | Adverse reaction rate | 7/7 | no serious | no serious | no serious | serious | na | would not | yes | no | high |
| CC:Clomiphene Citrate;LE:letrozole;HCG:Human Chorionic Gonadotropin;HMG:Urogonadotropin;CHM:Chinese Herbal Medicine;IVF-ET:In Vitro Fertilization-embryo Transfer;T:Testosterone;BMI:Body Mass Index;LH:Luteinizing Hormone;FSH:Follicle-stimulating Hormone;E2:Estradiol;BBT:Basal Body Temperature;HGF:Hepatocyte Growth Factor;VEGF:Vascular Endothelial Growth Facto;TNF-α:Tumor Necrosis Factor-α;OHSS:Ovarian Hyper-stimulation Syndrome;T:Total;R:RCT | | | | | | | | | | | | | |
